# ICOS^+^CD4^+^ T cells define a high susceptibility to anti–PD-1 therapy–induced lung pathogenesis

**DOI:** 10.1172/jci.insight.186483

**Published:** 2025-04-08

**Authors:** Mari Yokoi, Kosaku Murakami, Tomonori Yaguchi, Kenji Chamoto, Hiroaki Ozasa, Hironori Yoshida, Mirei Shirakashi, Katsuhiro Ito, Yoshihiro Komohara, Yukio Fujiwara, Hiromu Yano, Tatsuya Ogimoto, Daiki Hira, Tomohiro Terada, Toyohiro Hirai, Hirotake Tsukamoto

**Affiliations:** 1 Division of Clinical Immunology and Cancer Immunotherapy, Center for Cancer Immunotherapy and Immunobiology, Graduate School of Medicine, Kyoto University, Kyoto, Japan.; 2 Department of Clinical Pharmacology and Therapeutics, Kyoto University Hospital, Kyoto, Japan.; 3 Department of Immunology and Genomic Medicine, Center for Cancer Immunotherapy and Immunobiology,; 4 Department of Respiratory Medicine,; 5 Department of Rheumatology and Clinical Immunology, and; 6 Department of Urology, Graduate School of Medicine, Kyoto University, Kyoto, Japan.; 7 Department of Cell Pathology, Graduate School of Medical Science, Faculty of Life Sciences, Kumamoto University, Kumamoto, Japan.

**Keywords:** Aging, Autoimmunity, Immunology, Cancer immunotherapy, Mouse models, Tolerance

## Abstract

Managing immune-related adverse events (irAEs) caused by cancer immunotherapy is essential for developing effective and safer therapies. However, cellular mechanism(s) underlying organ toxicity during anti–PD-(L)1 therapy remain unclear. Here, we investigated the effect of chronological aging on anti–PD-(L)1 therapy–induced irAE-like lung toxicity, utilizing tumor-bearing aged mice. Anti–PD-(L)1 therapy facilitated ectopic infiltration of T and B cells, and antibody deposition in lungs of aged but not young mice. Adoptive transfer of aged lung–derived CD4^+^ T cells into TCR-deficient mice revealed that both pathogenic CD4^+^ T cells and an aged host environment were necessary for the irAE-inducible responses. Single-cell transcriptomics of lung-infiltrating cells in aged mice demonstrated that anti–PD-(L)1 therapy elicited ICOS^+^CD4^+^ T cell activation. Disruption of the ICOS-ICOSL interaction attenuated germinal center B cell differentiation and subsequent lung damage, which were overcome by local administration of IL-21 in the lungs of anti–PD-1 therapy–treated aged mice. Therefore, ICOS^+^CD4^+^ T cells elicited under an aged environment exacerbated aberrant immune responses and the subsequent lung dysfunction. Consistent with the findings from the mouse model, ICOS upregulation in CD4^+^ T cells was associated with later irAE incidence in patients with cancer. These finding will help development of useful strategies for irAE management in patients with cancer, many of whom are elderly.

## Abstract 2

Early induction of ICOS+CD4 T cells upon anti-PD-(L)1 therapy is associated with exacerbation of lung toxicity in aged animals and patients with cancer.

## Graphical abstract

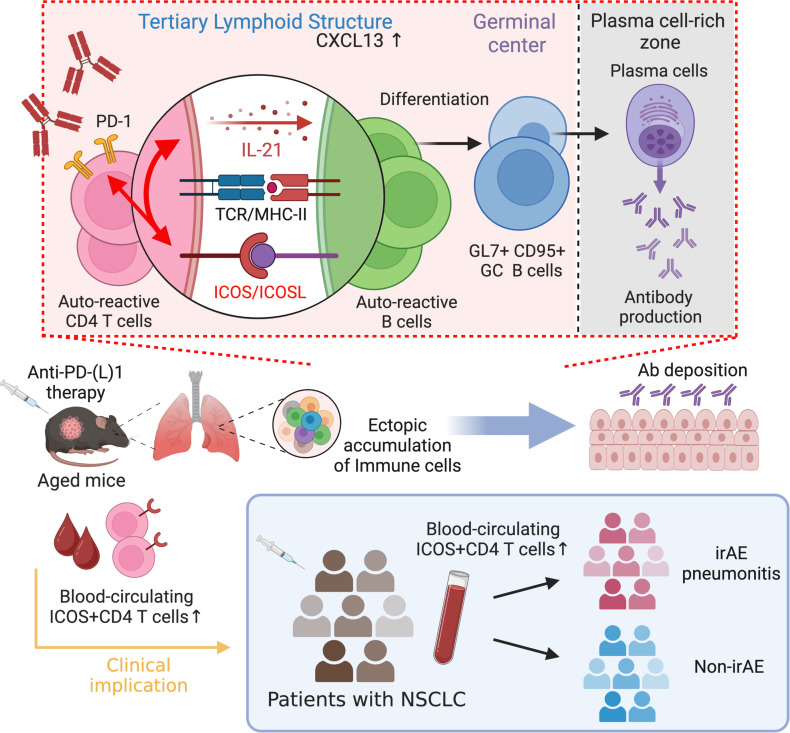

## Introduction

Outstanding progress has been made in anti–PD-(L)1 therapy combined with other immune checkpoint inhibitors (ICIs), radiation, and anticancer drugs (1). However, the additive effects of combination therapies have raised concerns about severe or even life-threatening organ toxicities, termed immune-related adverse events (irAEs) (2–4). Besides the increased risk of AEs, such as pneumonitis and kidney failure, in combination therapies with ICIs and anticancer drugs (4, 5), the difficulty in diagnosing which treatment induces AEs is also a clinically problematic issue. Severe irAEs lead to discontinuation of the therapy and therefore impede maximal clinical benefit, especially in elderly patients (6); in the worst cases with severe pneumonitis, they deprive opportunities for other treatments (7, 8). Additionally, given the potential detrimental effects of immunosuppressive drugs, including high-dose steroid used as symptomatic treatments on the effectiveness of ICIs (9, 10), more tailored irAE management strategies are required. Therefore, it is necessary to clarify the mechanism(s) underlying irAE development and to establish approaches that balance both enhancing antitumor immunity and limiting multiorgan toxicity.

Tumorigenesis is often associated with advanced age, and future projections indicate that more than half of the patients with cancer will be elderly individuals older than 65 years (11). Currently, a large proportion of patients receiving anti–PD-(L)1 therapy are elderly with age-associated changes in the immune system, termed immunosenescence (12, 13). In seeming contradiction to the attenuated defense response against infectious agents and cancers (13, 14), chronological aging is often the primary risk factor for autoimmune diseases, including auto-antibody–mediated (auto-Ab–mediated) organ dysfunction, with elevated levels of circulating IgG (15, 16). Moreover, anti–PD-(L)1 therapy–induced irAEs such as pneumonitis are more frequent in the elderly (17–19), which is often fatal and deserves special attention (20). However, older patients are sometimes underrepresented in clinical trials, despite them representing a major population in the clinical setting (21). The recent finding that aged mice were predisposed to anti–PD-(L)1 therapy–induced organ toxicities supports the age-associated increase in irAE incidence (22).

Based on clinical and pathological observations of diverse irAE manifestations, several possible immune responses underlying ICI-associated tissue damage have been postulated, and the causative immune reactions, such as cellular or humoral immunity, differ depending on each type of irAE (2). Since Abs against self-organs are likely prevalent in the pathogenesis of many irAEs (23, 24), defining the signals by which specific types of T cells trigger Ab-dependent autoimmune responses and elucidating where such signals are provided will allow the development of novel on-target strategies for irAE management. Recently, the B cell–attracting chemokine CXCL13 was identified as a potential trigger of irAE pathogenesis and dictates its organ specificity by promoting ectopic migration of immune cells and formation of tertiary lymphoid structure (TLS) in the corresponding organs (2, 22, 25). Of note, TLS localized in irAE-affected organs are highly involved in auto-Ab production, and TLS-related chemokines such as CXCL9, CXCL10, and CXCL13 demonstrate predictive value for ICI-induced irAEs (22, 25, 26). Although we previously showed that B cell depletion mitigated anti–PD-1 therapy–induced lung damage in aged mice (22), little is known about the detailed functionality of T cell subsets that contribute to the local pathophysiological cascade leading to auto-Ab production from B cells in irAEs.

In this study, we aimed to investigate the impact of chronological aging on anti–PD-(L)1 therapy–induced lung pathogenesis and the relevant immune responses using a tumor-bearing aged animal model. This model exhibited an elevated proportion of CD4^+^ T cells expressing inducible costimulator of T cells (ICOS), in both damaged lung and periphery upon anti–PD-(L)1 therapy. The interaction between ICOS and its ligand (ICOSL) promotes specific Ab production through promoting responses of T follicular helper (Tfh) cells, subsequent B cell differentiation, and formation of functional germinal centers (GCs) in secondary lymphoid tissue in the contexts of infection and autoimmune diseases (27, 28). However, its role in the pathogenicity of irAEs has yet to be determined. We hereafter demonstrated that ICOS-ICOSL interaction within the TLS contributed to the deterioration of anti–PD-(L)1 therapy–induced lung pathogenesis.

## Results

### Anti–PD-1 therapy induces CD4^+^ T cell–dependent lung damage in tumor-bearing aged mice.

**Figure 1:**
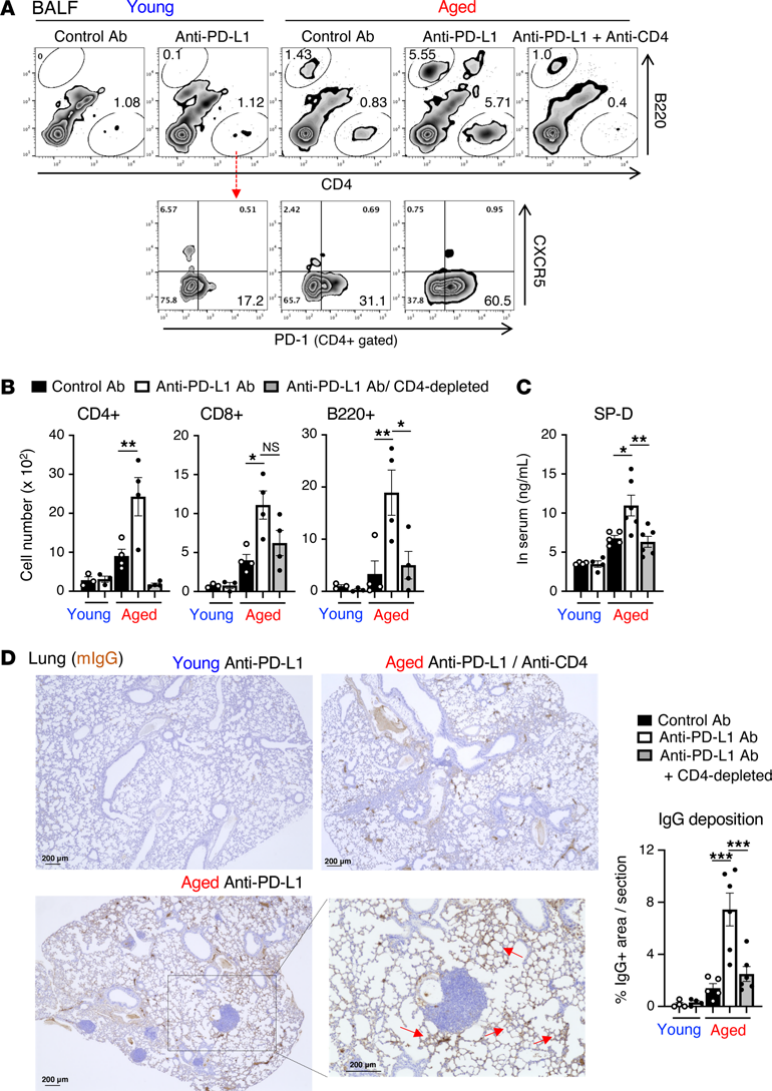
CD4^+^ T cells are necessary for the development of anti–PD-L1 therapy–induced lung damage. MC38-bearing young or aged mice were treated with anti–PD-L1 Ab 3 times. CD4^+^ T cells were depleted from aged mice by administering anti-CD4 Ab. (**A** and **B**) Numbers of B and T cells in BALF and their PD-1 or CXCR5 expression were analyzed. Representative plots (**A**) and absolute number of indicated populations (**B**) are shown. NS, not significant. (**C**) SP-D levels in the serum from indicated mice were measured. (**D**) Representative lung sections stained with anti–mouse IgG Ab (mIgG) are shown. Scale bars: 200 μm. The right panel of **D** indicates the percentage IgG^+^ area in lung sections. Data are representative of 3 independent experiments with similar results and presented as mean ± SEM (*n* = 3–6). *P < 0.05; **P < 0.01; ***P < 0.001 by 1-way ANOVA followed by Tukey-Kramer post hoc test.

Since anti–PD-(L)1 therapy has been shown to cause toxicities in several organs, including the lung in tumor-bearing aged mice, but not in young counterparts (22), we sought to elucidate the involvement of T cells and B cells in anti–PD-(L)1 therapy–induced lung pathogenesis in aged mice. In contrast with the case of young mice, substantial increases in B220^+^ B cells and CD8^+^ and CD4^+^ T cells were detected in bronchoalveolar lavage fluid (BALF) of tumor-bearing aged mice, particularly when they were treated with anti–PD-L1 therapy (Figure 1, A and B). In aged BAL CD4^+^ T cells, anti–PD-L1 therapy induced upregulation of PD-1 expression, but not that of the Tfh marker CXCR5 (Figure 1A). Depletion of CD4^+^ T cells in aged mice impeded the accumulation of B cells, even with anti–PD-L1 Ab administration (Figure 1B). In accordance herewith, CD4^+^ T cell depletion improved anti–PD-L1 Ab–induced lung abnormalities in aged mice, as indicated by elevated leakage of surfactant protein D (SP-D) into circulating blood from injured lungs, indicative of lung damage (Figure 1C), and aberrant IgG deposition in the lung tissues of anti–PD-1 therapy–treated mice (Figure 1D). Concomitantly, immune cells including T cells ectopically aggregated in the lungs of aged mice (Supplemental Figure 1A). Such aggregates have been demonstrated to have the features of TLS (22).

The involvement of B cells in this lung pathogenesis was confirmed by inefficient TLS formation (Supplemental Figure 1A; supplemental material available online with this article; https://doi.org/10.1172/jci.insight.186483DS1) (22) and decreased accumulation of CD4^+^ T cells in the lungs when B cells were depleted (Supplemental Figure 1B). However, B cell depletion did not alter the antitumor effect of PD-1 blockade (Supplemental Figure 1C). These results suggest that an interplay and reciprocal activation between B cells and CD4^+^ T cells are responsible for anti–PD-(L)1 therapy–induced lung pathogenesis via aberrant IgG deposition. The ectopic immune cell accumulation and SP-D leakage were observed to a certain extent in nontreated aged mice, and exacerbated by anti–PD-1 therapy even in tumor-free aged mice (Supplemental Figure 2A). Anti–PD-1 therapy elicited higher expression of chronic obstructive pulmonary disease–associated (COPD-associated) genes and some inflammatory signatures in aged mice (Supplemental Figure 2B). These observations imply that aged mice have fundamental prelesions predisposing to the lung damage and inflammation, which is worsened by anti–PD-(L)1 therapy.

### CD4^+^ T cells promote GC B cell differentiation responsible for lung pathogenesis.

**Figure 2:**
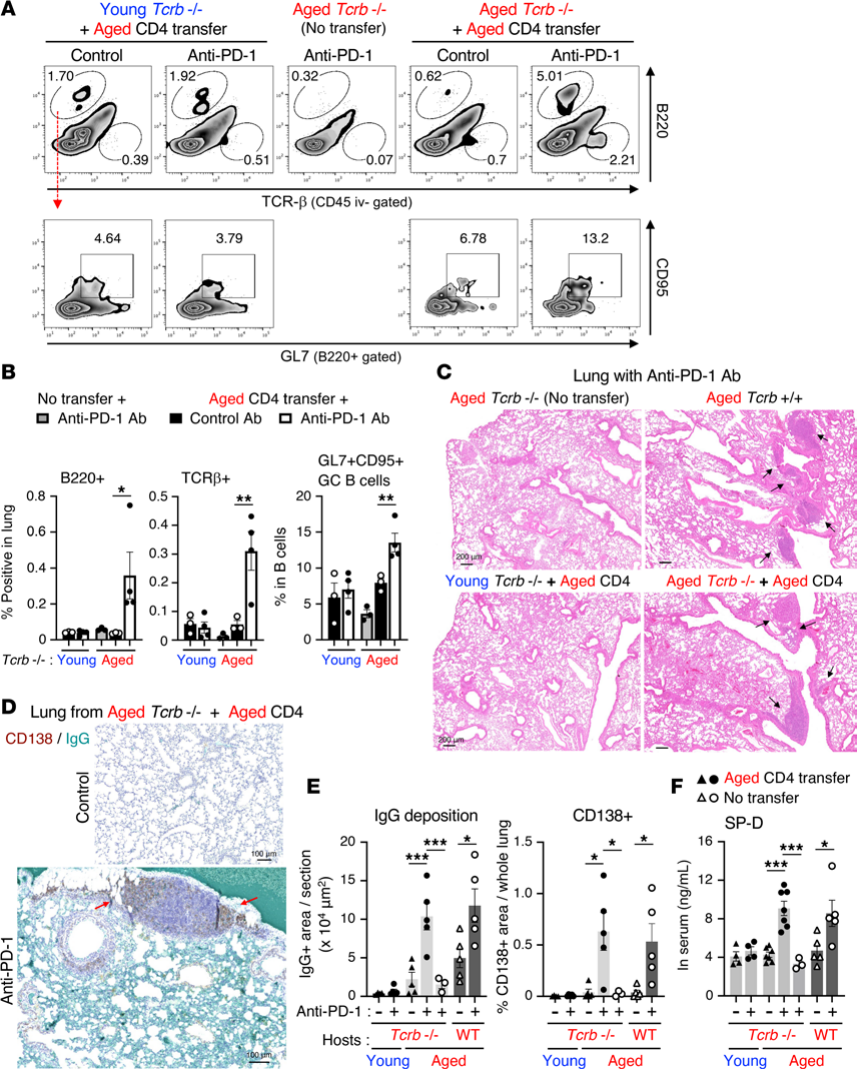
Reconstitution of pathogenic CD4^+^ T cells induces lung damage in T cell–deficient aged mice, but not in young mice. CD4^+^ T cells isolated from the lungs of anti–PD-1 therapy–treated aged mice were transferred into TCR-deficient young or aged mice, which then were treated with anti–PD-1 Ab. (**A** and **B**) To discriminate the lung-infiltrating cells from blood-circulating cells, anti-CD45 Ab was injected intravenously before harvesting the lung cells. Lung-infiltrating CD45 iv-staining negative populations (iv-) cells were analyzed for infiltration of donor TCRβ^+^ cells and B cells, and their CD95^+^GL7^+^ GC subset. Representative dot plots (**A**) and frequencies of the indicated populations (**B**) are shown. (**C**–**E**) Representative H&E-stained sections of lung (**C**) and IgG deposition (**D**) are shown. Scale bars: 200 μm (**C**) and 100 μm (**D**). The left and right panels in **E** indicate the percentage of IgG^+^ and CD138^+^ area in lung sections, respectively. (**F**) SP-D levels in the serum from indicated mice were measured. Data are representative of more than 2 experiments and presented as mean ± SEM (*n* = 3–7). *P < 0.05; **P < 0.01; ***P < 0.001 by 1-way ANOVA followed by Tukey-Kramer post hoc test.

We hypothesized that the irAE-like symptoms in aged mice were induced by CD4^+^ T cell–mediated promotion of Ab production by B cells. To test this hypothesis, we established aged mice deficient in T cell receptor β chain (TCRβ; normally expressed by αβ T cells), which causes a reduction in αβ T cells, and assessed lung-infiltrating lymphocytes that were discriminated from blood-circulating cells stained via in vivo intravenous (iv) injection of anti-CD45 Ab (CD45 iv) before harvesting the lung (Supplemental Figure 2C). In contrast with the case of aged wild-type (WT) mice (Figure 1), the ectopic infiltration of CD45 iv- B cells was completely abolished in aged TCRβ-deficient mice, regardless of anti–PD-1 therapy (Figure 2, A and B). Moreover, to validate the role of CD4^+^ T cells in the lung pathogenesis, aged lung-infiltrating CD4^+^ T cells were transferred into young or aged TCRβ-deficient mice, and their lung pathology was assessed. Unexpectedly, when the young TCRβ-deficient mice were utilized as hosts, B cells and donor aged CD4^+^ T cells did not efficiently infiltrate or expand in the lungs despite anti–PD-1 therapy (Figure 2, A and B). In contrast, anti–PD-1 therapy elicited substantial accumulation of B cells and donor CD4^+^ T cells in the lung of aged TCRβ-deficient mice. Of note, a portion of these B cells ectopically differentiated into GL7^+^CD95^+^ GC B cells only in an aged environment (Figure 2, A and B).

Ectopic immune cell infiltration in the lung coincided with histological detection of local lymphocytic aggregates in anti–PD-1 therapy–treated WT aged mice (Figure 2C). As in WT aged mice, such TLS-like lymphocytic aggregates were observed in the lungs of aged TCRβ-deficient mice only when reconstituted with aged-lung-derived CD4^+^ T cells and treated with anti–PD-1 therapy. On the other hand, such TLS-like lymphocytic aggregates were not observed in young TCRβ-deficient mice even after transfer of aged CD4^+^ T cells, implying that aged lung environmental factors also served as a driving force for ectopic immune responses. Moreover, IHC of lung tissues using an anti–mouse IgG Ab also revealed that anti–PD-1 therapy and reconstitution with pathogenic aged CD4^+^ T cells in an aged environment induced obvious IgG deposition in host lung tissues and concomitant CD138^+^ plasma cells around the TLS (Figure 2, D and E). Accompanied with these immune responses, anti–PD-1 therapy significantly increased the levels of blood SP-D in CD4^+^ T cell–complemented TCRβ-deficient aged mice, compared with those in young or nontransferred aged TCRβ-deficient hosts (Figure 2F). The levels of serum SP-D at steady state in aged mice were also reduced by T cell deficiency. Taken together, these results suggest that the PD-1–PD-L1 interaction maintains tolerance of the pathogenic T cell–dependent Ab response against aged lung tissues. More importantly, CD4^+^ T cells enriched in both the lung and an aged environment served as critical determinants for the differentiation of B cells into Ab-secreting cells during anti–PD-1 therapy–induced lung pathogenesis.

### ICOS-expressing effector CD4^+^ T cells are enriched in damaged lungs of anti–PD-1 Ab–treated aged mice.

**Figure 3:**
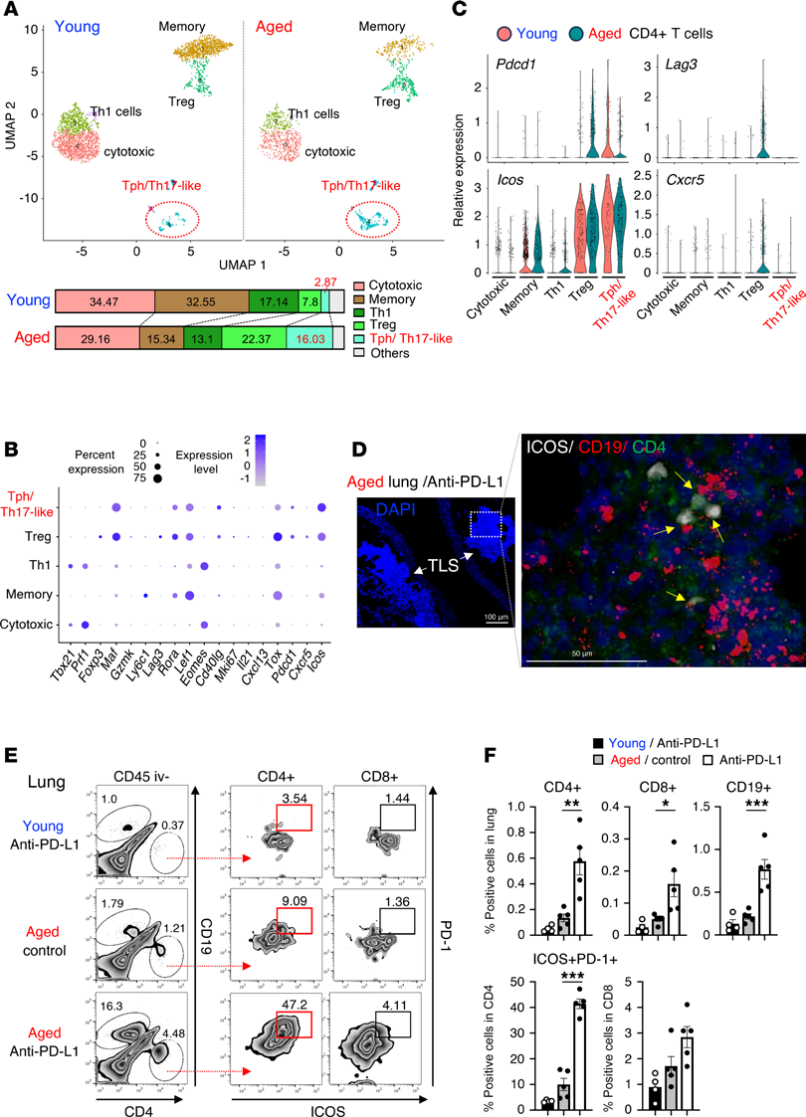
ICOS-expressing effector CD4^+^ T cells excessively accumulate in lung tissues of aged mice. MC38-bearing young or aged mice were treated with anti–PD-L1 Ab. (**A**–**C**) Lung-infiltrating CD45^+^ cells were isolated from MC38-bearing young or aged mice that were treated with anti–PD-L1 Ab, and then were analyzed by scRNA-seq. Cluster analysis was performed and plotted by UMAP dimensionality reduction. Subclustering was performed on the data from the *Cd4*^+^ T cell populations in young and aged mice, and lower bar plots in **A** indicate their frequencies. Dot plots shown in **B** represent the expression of canonical marker genes across the indicated *Cd4*^+^ subclusters. Relative expression of indicated genes across subclustered populations from young and aged mice are shown in **C**. (**D**) Representative images of DAPI staining (left) and in situ RNA hybridization (right; white, ICOS; red, CD19; green, CD4) in lung from aged mice are shown. Scale bars: 100 μm (left) and 50 μm (right). (**E**) Representative dot plots of CD45 iv- cell populations within the lungs are shown. (**F**) Frequencies of indicated lung-infiltrating CD45^–^ cell populations were assessed in mice treated with indicated Abs. Data are represented as mean ± SEM. *P < 0.05, **P < 0.01, ***P < 0.001 by 1-way ANOVA followed by Tukey-Kramer post hoc test.

To clarify which CD4^+^ T cell subsets were involved in the lung pathogenesis induced by anti–PD-(L)1 therapy in aged mice, lung tissues from anti–PD-L1 therapy–treated young or aged mice were analyzed for the transcriptomic profiles of CD45^+^ immune cells using single-cell RNA sequencing (scRNA-seq). Because many lymphocyte populations were presumably derived from circulating blood in the lung, there were few differences in immune cell components between young and aged groups (Figure 3A). However, subclustering of the CD4^+^ T cell population and gene expression profiling of each subpopulation of young or aged mice revealed that the *Pdcd1*^+^*Icos*^+^*Cd69*^+^*Cxcr5*^–^*Rora*^+^ effector *Cd4*^+^ T cell cluster, which comprised T peripheral helper (Tph)/Th17-like cells, was more abundant in the lungs of aged mice, as compared with young mice treated with anti–PD-L1 therapy (Figure 3, A and B). The expression level of *Icos* in this cluster as well as in *Foxp3*^+^*Lag3*^+^ regulatory T (Treg) cells was higher than those in the other clusters in aged lung (Figure 3C). Moreover, in situ RNA hybridization analyzing the spatial distribution of *Icos* expression of aged lungs treated with anti–PD-L1 therapy showed that *Icos*-expressing *Cd4*^+^ T cells closely interacted with *Cd19*^+^ B cells within the TLS-like lymphocytic aggregates (Figure 3D and Supplemental Figure 3). However, we did not rule out the possibility that these *Icos*^+^ T cells were Treg cells.

Because lung CD45^+^ cells analyzed by scRNA-seq seemed to be contaminated with blood cells from the circulation, we validated surface expression of ICOS in lung-infiltrating T cells using flow cytometry (Figure 3E). Lung-infiltrating T and B cells that can be discriminated from blood-circulating cells labeled with intravenously injected anti-CD45 Ab were increased upon anti–PD-L1 Ab administration in aged mice. Of note, Foxp3^+^ Tregs did not efficiently infiltrate into the lung compared with effector CD4^+^ T cells (Supplemental Figure 4A), while tumor-infiltrating Treg cells highly expressed ICOS regardless of host age or anti–PD-L1 therapy (Supplemental Figure 4B). In line with the transcriptomic analyses, the expression of both ICOS and PD-1 was significantly upregulated in aged lung-infiltrating CD4^+^ T cells (Figure 3, E and F). The majority of lung-infiltrating ICOS^+^CD4^+^ T cells expressed B cell lymphoma 6 (Bcl-6) (Supplemental Figure 4C). In contrast, ICOS was not expressed in lung-infiltrating CD8^+^ T cells (Figure 3E) or IL-17–producing CD4^+^ T cells in damaged lungs (Supplemental Figure 4D). These observations suggest that accumulation of a unique ICOS^+^CD4^+^ effector T cell population became prominent in damaged lungs of aged mice in response to anti–PD-1 therapy, which prompted us to investigate the contribution of ICOS to the lung pathogenesis.

### ICOS-ICOSL interaction contributes to anti–PD-1 therapy–induced lung pathogenesis.

**Figure 4:**
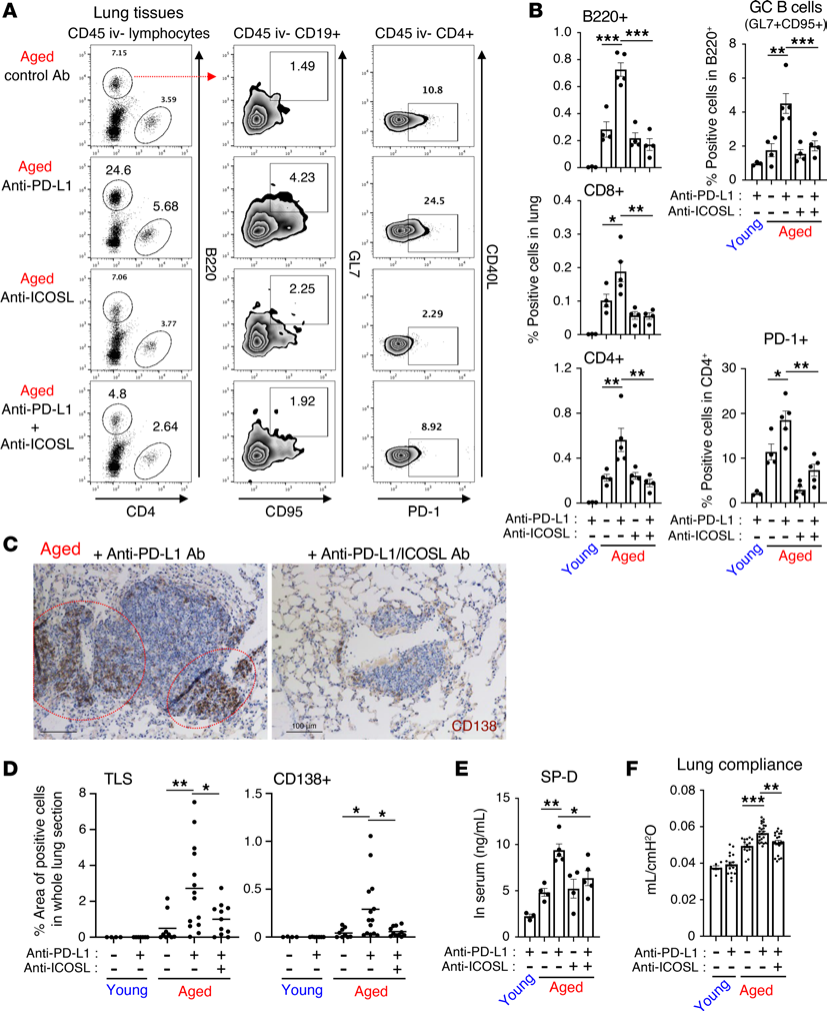
Abrogation of ICOS-ICOSL interaction with anti-ICOSL Ab attenuates the development of lung injury caused by anti–PD-(L)1 Ab in aged mice. Tumor-bearing young or aged mice were administered with anti–PD-L1 Ab. Anti-ICOSL Ab was injected to block the ICOS-ICOSL interaction. (**A** and **B**) Representative dot plots of CD45 iv- lung-infiltrating cell populations from MC38-bearing mice (**A**) and their frequencies (**B**) are shown. (**C** and **D**) Representative images of CD138 IHC in the lung of tumor-bearing aged mice (**C**), and the percentage area of TLS (left) and CD138^+^ plasma cells (right) in each lung section (**D**) are shown. Scale bars: 100 μm. (**E**) The levels of SP-D in the serum are shown. (**F**) Lung dysfunction in tumor-bearing mice was assessed by mechanical ventilation system. Data are representative of 3 independent experiments with similar results and presented as mean ± SEM (*n* = 4–8). *P < 0.05; **P < 0.01; ***P < 0.001 by 1-way ANOVA followed by Tukey-Kramer post hoc test.

We next assessed the role of the ICOS-ICOSL interaction in anti–PD-1 therapy–induced lung pathogenesis. Concomitant administration of anti–PD-1 Ab and anti-ICOSL Ab to disrupt the ICOS-ICOSL interaction decreased the frequencies of lung-infiltrating CD4^+^ T cells and, even more so, B cells, whereas treatment with anti-ICOSL Ab alone did not alter their frequencies (Figure 4, A and B). Moreover, the differentiation of lung-infiltrating B cells into GC B cells was also blunted by anti-ICOSL Ab coadministration. The downregulation of PD-1 expression in CD4^+^ T cells from aged mice treated with both anti-ICOSL Ab and anti–PD-L1 Ab also implied ICOS-dependent activation of CD4^+^ T cells (Figure 4, A and B). These results suggest that ICOS stimulation is likely essential for the prosurvival signals in ICOS^+^CD4^+^ T cells via ICOSL engagement and subsequent helper activity of T cells toward B cells within the lungs. Consistent herewith, TLS formation and accumulation of CD138^+^ plasma cells existed outside TLS in the lungs of anti–PD-(L)1 therapy–treated aged mice were substantially abolished by combined blockade of the ICOS-ICOSL interaction (Figure 4, C and D, Supplemental Figure 5A). Furthermore, the ICOSL blockade–mediated immunological changes were accompanied by decreased leakage of SP-D in the blood from injured lung (Figure 4E) and improved the lung compliance, indicative of pulmonary dysfunction in respiratory function tests (Figure 4F). Pathologically, in aged mice, ICOSL blockade also restored the anti–PD-1 therapy–induced increase in alveolar size (Supplemental Figure 5B), which is associated with decline in lung function (29). From these results, it was assumed that ICOS-mediated CD4^+^ T cell activation exacerbated lung dysfunctions such as geriatric emphysema that was a risk predisposition for ICI-induced pneumonitis (30).

ICOS-mediated helper activity for B cell responses, including GC B cell differentiation and subsequent exacerbation of lung pathogenicity in aged B6 mice, were generalized by similar findings in colon cancer–bearing aged BALB/c mice treated with anti–PD-1 therapy (Supplemental Figure 6, A and B). ICOS-ICOSL interaction contributed to exacerbation of the irAE autoimmune response by promoting CD4^+^ T cell activation and subsequent B cell differentiation into Ab-secreting cells in damaged lungs (Supplemental Figure 6C). In contrast with the effects on lung pathogenesis, combined treatment with anti-ICOSL Ab did not modulate PD-(L)1 blockade–induced antitumor effects in either young or aged mice (Supplemental Figure 6D and Supplemental Figure 7A) and did not alter the frequencies of IFN-γ–producing CD4^+^ and CD8^+^ T cells (Supplemental Figure 7B). Taken together, these results suggest that blockade of ICOS’s interaction with ICOSL in CD4^+^ T cells mitigates anti–PD-(L)1 therapy–induced lung pathogenesis regardless of genetic background, type of Abs disrupting the PD-1–PD-L1 interaction, and their antitumor efficacy.

### ICOS-dependent IL-21 induction is crucial for anti–PD-1 therapy–induced lung damage.

**Figure 5:**
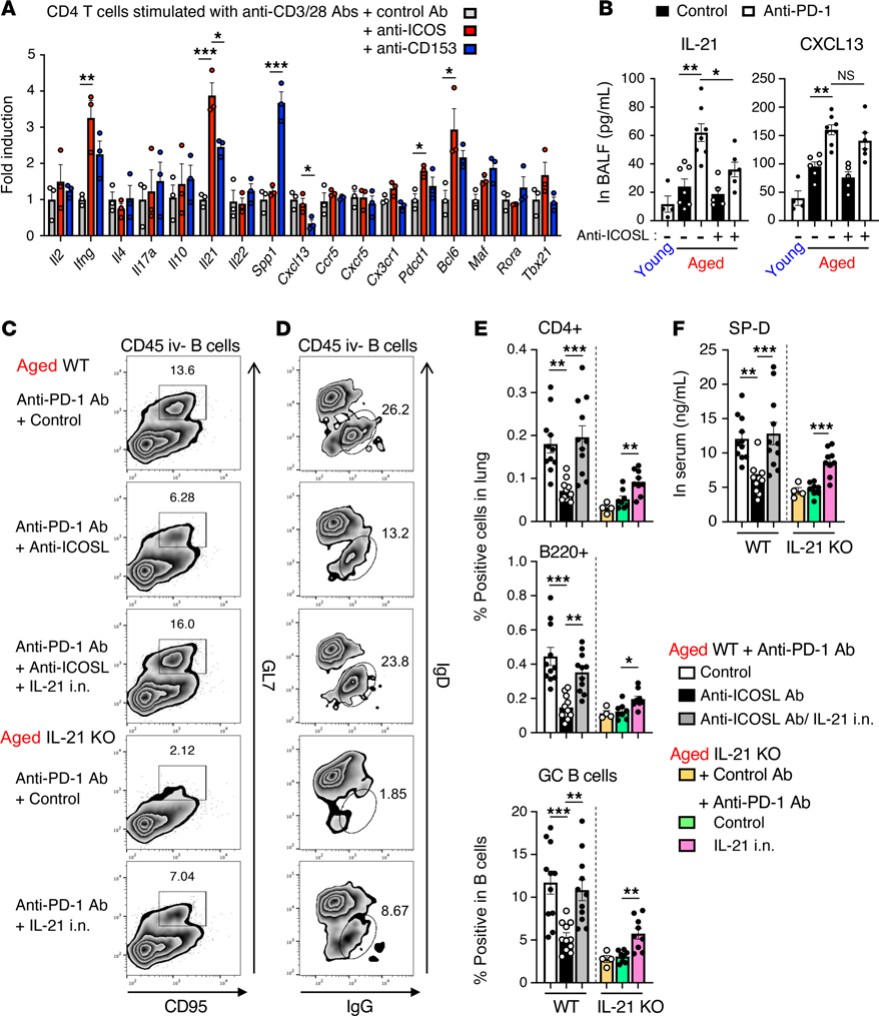
IL-21 is indispensable for ICOS stimulation–mediated exacerbation of anti–PD-1 therapy–induced lung pathology. (**A**) CD4^+^ T cells were isolated from the lungs of aged mice treated anti–PD-1 therapy and then were stimulated with anti-CD3/anti-CD28 Abs together with anti-ICOS or anti-CD153 agonistic Ab ex vivo. Fold inductions of indicated mRNA expression relative to that in control Ab-stimulated cells are shown. (**B**) Concentration of IL-21 or CXCL13 in BALF isolated from young or aged mice with indicated treatment was measured. Data are mean ± SEM (*n* = 3–6). One-way ANOVA followed by Tukey’s post hoc test was used. (**C**–**F**) WT or IL-21–deficient aged mice were treated with anti–PD-1 and anti-ICOSL Abs, and inoculated intranasally (i.n.) with recombinant IL-21. Representative plots of CD95^+^GL7^+^ cells (**C**) and IgG^+^ cells (**D**) among lung-infiltrating B cells, their frequencies (**E**), and serum concentrations of SP-D (**F**) are shown. Data are mean ± SEM, and the results from 2 independent experiments were combined (n = 4–6 per experiment). Then, 1-way ANOVA followed by Tukey-Kramer post hoc test was conducted for aged WT or IL-21–KO groups separately. *P < 0.05; **P < 0.01; ***P < 0.001.

To further assess the mechanisms involved, we examined the expression of several factors in lung-infiltrating CD4^+^ T cells from anti-–PD-1 therapy–treated aged mice, with or without ex vivo ICOS stimulation (Figure 5A). This analysis verified the intrinsic ability of ICOS stimulation to significantly upregulate the expression of *Il21* and *Pdcd1* (PD-1) in aged lung-infiltrating CD4^+^ T cells. ICOS stimulation also led to the upregulation of Bcl-6, which is a critical transcription factor for IL-21 (31), but not other subset-related transcription factors. Although the expression of *Ifng* was also induced ex vivo (Figure 5A), its deficiency did not affect the anti–PD-1 therapy–induced lung pathogenesis (22), implying less importance of IFN-γ in lung pathogenesis. In contrast with ICOS stimulation, stimulation with an agonistic Ab against CD153, which has been reported to help Ab responses in aged mice (32), did not efficiently induce these genes (Figure 5A), despite substantial expression of CD153 in aged lung-infiltrating CD4^+^ T cells (Supplemental Figure 8, A and B). These results suggested a direct and crucial regulatory role of ICOS signaling for the induction of IL-21, Bcl-6, and PD-1 in aged CD4^+^ T cells during anti–PD-(L)1 therapy–mediated irAE exacerbation. Indeed, anti–PD-1 therapy increased the levels of IL-21 in BALF of tumor-bearing aged mice, which was disrupted by ICOSL blockade (Figure 5B), and lung-infiltrating ICOS^+^CD4^+^ T cells in anti–PD-1 Ab–treated aged mice had an ability to produce IL-21 (Supplemental Figure 8C). On the other hand, ICOSL blockade did not affect the elevated levels of CXCL13 in the BALF (Figure 5B). These data are in line with the in vitro results and were reminiscent of the role of ICOS in facilitating the mutual activation of T and B cells and the formation and maintenance of GC cells.

To further clarify the mechanistic action of ICOS^+^CD4^+^ T cells in anti–PD-(L)1 therapy–induced irAE-like lung pathogenesis, intranasal administration of IL-21 in WT or IL-21–deficient aged mice was performed together with coblockade of PD-1 and ICOS signals. As shown in Figure 5, C and D, the inhibitory effects of ICOSL blockade on anti–PD-1 therapy–induced accumulation of B cells and GC B cell differentiation were significantly reversed by replenishment of recombinant IL-21 in WT aged mice. This was also the case with IL-21–deficient aged mice. While ICOSL blockade inhibited the induction of IgG-expressing GC B cells in anti–PD-1 therapy–treated aged WT mice, intranasal administration of IL-21 exacerbated these responses in anti-ICOSL Ab–treated WT or IL-21–deficient aged mice (Figure 5, C–E). Accompanied with these immune responses in the lung, the prominent increase in leakage of SP-D into circulating blood in anti–PD-1 therapy–treated aged mice was abrogated by ICOSL blockade or IL-21 deficiency (Figure 5F). Exogenous IL-21 administration abrogated the beneficial effects of ICOSL blockade or IL-21 deficiency on the aberrant increase in serum SP-D. Taken together, these results revealed that local IL-21 induction via ICOS signaling is responsible for the differentiation into pathogenic Ab-producing B cells and the subsequent development of irAE-like pathology.

### The proportion of peripheral ICOS^+^CD4^+^ T cells is associated with irAE incidence in patients with cancer.

**Figure 6:**
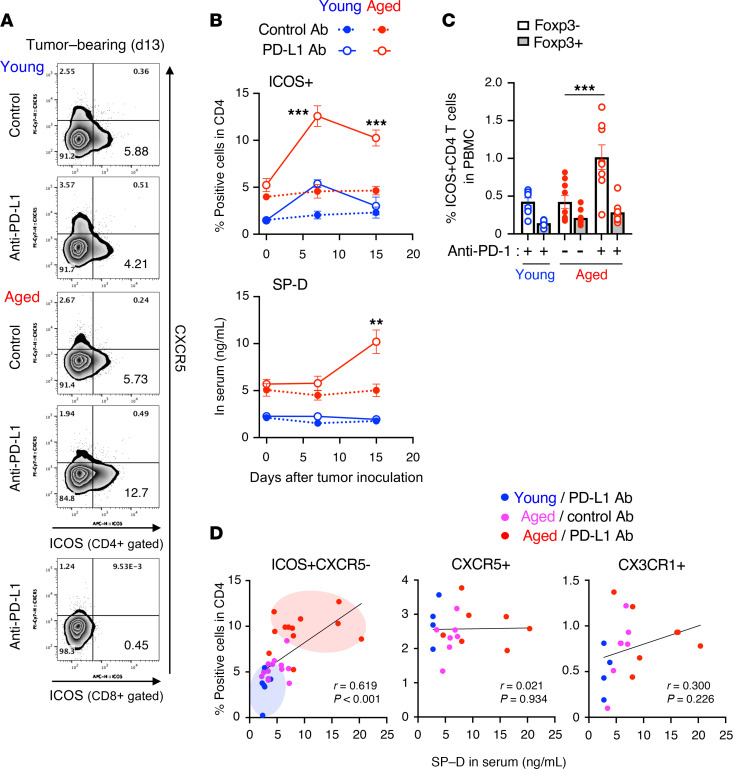
Anti–PD-L1 therapy causes ICOS^+^CD4^+^ T cells to be retained in the periphery of MC38-bearing aged mice. MC38-bearing young or aged mice were treated with control or anti–PD-(L)1 Ab. (**A** and **B**) The representative dot plots of blood-circulating CD4^+^ T cells 13 days after tumor inoculation (**A**), and kinetic changes in frequencies of the indicated populations (**B**) are shown (*n* = 5–6). (**C**) Frequencies of Foxp3^–^ or Foxp3^+^ICOS^+^CD4^+^ T cells in the peripheral blood were analyzed (*n* = 8–9). **P < 0.01, ***P < 0.001 by 1-way ANOVA followed by Tukey’s post hoc test. (**D**) Correlation between the frequencies of indicated populations and the concentration of SP-D is shown. Simple linear regression analysis was conducted. Data are mean ± SEM from more than 2 independent experiments with similar results.

Considering the role of ICOS^+^CD4^+^ T cells in irAE development, it is possible that they have predictive value for irAE development. However, in clinical settings, it is difficult to assess tissue-infiltrating cells in irAE-affected organs of patients. Therefore, we examined ICOS expression in blood-circulating CD4^+^ T cells and its association with lung pathology. As observed in lung tissues, ICOS expression in peripheral CD4^+^ T cells was elevated in circulating blood from aged mice, but not from young mice, immediately after initiation of anti–PD-1 therapy (Figure 6, A and B, and Supplemental Figure 8D). This preceded the increase in SP-D levels. On the other hand, the proportion of Foxp3^+^ICOS^+^CD4^+^ Treg cells did not increase in the periphery (Figure 6C). More importantly, the frequencies of ICOS^+^CD4^+^ T cells in the blood correlated with the serum SP-D levels in injured lungs 15 days after tumor inoculation (Figure 6D).

**Figure 7:**
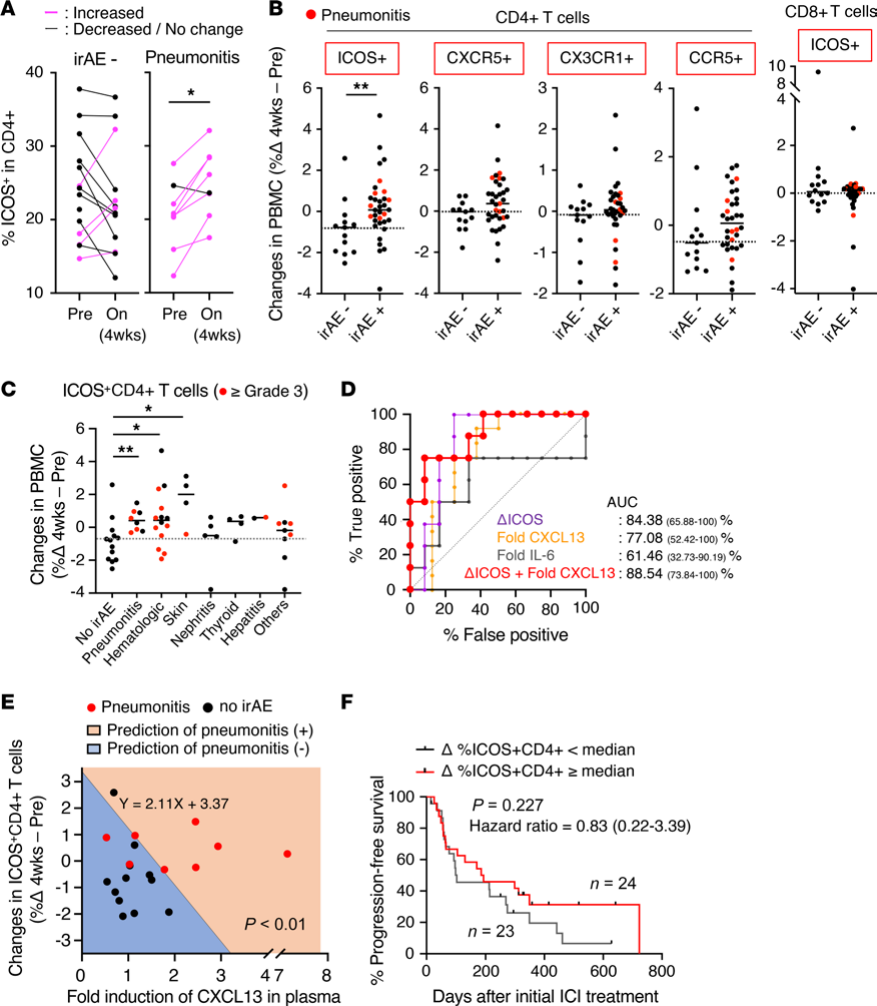
An increase in peripheral ICOS^+^CD4^+^ T cells is associated with the incidence of anti–PD-(L)1 therapy–induced irAEs in patients with NSCLC. (**A**) The frequency of ICOS^+^ cells in peripheral CD4^+^ T cells was analyzed before (Pre) and 4 weeks after initial anti–PD-(L)1 therapy (On) in patients with (*n* = 8) or without (*n* = 13) the irAE pneumonitis. *P < 0.05 by Wilcoxon’s signed-rank test. (**B**) Changes in the frequencies of indicated CD4^+^ or CD8^+^ T cell subsets during 4 weeks of anti–PD-(L)1 therapy in patients with (*n* = 34) or without (*n* = 13) irAEs, were analyzed. Red dots indicate the values for patients with pneumonitis. (**C**) Changes in the frequency of ICOS^+^CD4^+^ T cells in patients with indicated irAEs are shown. “Others” includes symptoms with infusion reaction, fever, adrenal irAEs, carditis, diarrhea, hypophysitis, and neurologic irAEs. *P < 0.05; **P < 0.01 by Mann-Whitney *U* test. (**D**) Univariate (ΔICOS, fold change in CXCL13 or IL-6) and multivariate (ΔICOS plus changes in CXCL13) ROC analyses of the predictive values for pneumonitis development. (**E**) Distribution of indicated values in the combined predictive model for pneumonitis incidence in patients with pneumonitis (*n* = 8) or without (*n* = 12) irAEs. Adonis test based on the Bray-Curtis distance was used. (**F**) Kaplan-Meier plots of progression-free survival of patients with NSCLC stratified according to the median value of the change in peripheral ICOS^+^CD4^+^ T cells are shown. Log-rank (Mantel-Cox) test and univariate Cox proportional hazards analyses were performed.

To validate the findings from the preclinical aged mouse model, we evaluated the frequency of ICOS^+^CD4^+^ T cells in peripheral blood mononuclear cells (PBMCs) from patients with non–small cell lung cancer (NSCLC) before and 4 weeks after anti–PD-(L)1 therapy (Supplemental Figure 9A). Patient information is described in Supplemental Table 1. As reported in previous retrospective studies (33), pneumonitis was an irAE observed at a high frequency (16%) in patients with NSCLC in our cohort. While the proportions of both CD4^+^ T cells and CD8^+^ T cells were not altered by the therapy (Supplemental Figure 9B), the expression of ICOS in peripheral CD4^+^ T cells was significantly upregulated in patients with the irAE pneumonitis, but not in those without any irAEs (Figure 7, A and B). All patients who received ICIs combined with chemotherapy were treated with dexamethasone as premedication. The use of the immunosuppressive drugs had no noticeable effect on irAE development (Supplemental Tables 1–3) or frequencies of ICOS^+^CD4^+^ T cells (Supplemental Figure 9C). In 31 of 49 patients (61.2%), including more than 85% of pneumonitis cases, irAEs had not developed 4 weeks after initial anti–PD-(L)1 therapy (Supplemental Figure 9E). The increase in the frequency of ICOS^+^CD4^+^ T cells in the blood was characteristic not only of patients with pneumonitis but also of those with hematological and skin irAEs (Figure 7, B and C, and Supplemental Table 2). However, attempts to classify ICOS expression in irAEs based on treatment regimens were limited by the small sample size (Supplemental Figure 9D). Indeed, a significant increase in ICOS^+^CD4^+^ T cells was observed in patients with irAEs who received a combination of ICI and chemotherapy, but not in the ICI monotherapy–treated subgroup, probably due to the limited number of patients. In contrast with ICOS expression, changes in frequencies of CXCR5^+^, CX3CR1^+^, or CCR5^+^ CD4^+^ T cells, which have been reported to be expressed in Tfh or Tph cells (34), or ICOS^+^CD8^+^ T cells were comparable between patients with and without irAEs (Figure 7B). Moreover, the levels of CXCL13, a B cell–attracting chemokine, tended to be increased in plasma of patients with pneumonitis but not in that from patients without any irAEs (Supplemental Figure 9F and Supplemental Table 2) (22), and the inflammatory cytokine IL-6 has been reportedly involved in anti–PD-(L)1 therapy–induced pneumonitis (35). We thus investigated the predictive value of these factors for development of irAEs, including pneumonitis, using univariate and multivariate receiver-operating characteristic (ROC) analyses (Supplemental Table 4). An increased frequency of ICOS^+^CD4^+^ T cells was a better predictor for pneumonitis development than those of CXCL13 or IL-6 levels (Figure 7D and Supplemental Table 3), and combined assessment of both changes in ICOS^+^CD4^+^ T cells and CXCL13 levels had an even greater predictive value for later development of pneumonitis (Figure 7D and Supplemental Table 5).

Based on the cutoff value (0.56) obtained from multivariate ROC analysis in this combined evaluation and the propensity score for each patient, a prediction equation for the development of the irAE pneumonitis was established (Supplemental Table 5). When the corresponding values from patients with or without pneumonitis were applied to the model with this predictive equation, patient groups with the irAE pneumonitis and the non-irAE group could be statistically distinguished as distinct components (sensitivity; 75%, specificity; 92%, Adonis test *P* < 0.01) (Figure 7E), suggesting the predictive value of combined evaluation of CXCL13 and ICOS for the development of the irAE pneumonitis. On the other hand, there was no significant difference in the antitumor effect of ICIs evaluated by duration of progression-free survival (PFS) (Figure 7F) or by overall response (Supplemental Figure 10A), when stratified according to the changes in ICOS^+^CD4^+^ T cells. While the changes in CXCL13 levels also had no effect on antitumor effects of ICIs (Supplemental Figure 10B), the irAE incidence was associated with longer period of their overall survival (OS) (Supplemental Figure 10C).

## Discussion

irAE incidence remains largely unpredictable and poorly discriminated among diverse disease types. The identification of irAE-prone patients and a mechanism-based classification of irAEs will enable precise, patient-tailored treatment and broaden the patient population that can benefit from cancer immunotherapy. Our current results from aged mice and other studies utilizing patient databases suggest that chronological aging may be a risk factor for developing irAEs during anti–PD-(L)1 therapy; however, this assumption remains controversial (17, 18, 36). Considering the difficulty in analyzing age-related immunological changes in human specimens with differences in exposed environmental factors and their genetic backgrounds, we took advantage of an experimental model with tumor-bearing aged mice to extract factors that dictate the development of irAEs in the current study. Our experimental model of anti–PD-(L)1 therapy–induced lung toxicity provided, for the first time to our knowledge, mechanistic insights into age-associated ICOS^+^CD4^+^ T cells underlying local organization of B cell–mediated auto-Ab responses, which have been expected to be involved in the pathogenesis of irAEs (2, 23, 24). This perspective is in line with the association of chronological aging with auto-Ab–dependent tissue dysfunction in some autoimmune diseases (15, 16). Furthermore, a notable increase in ICOS^+^CD4^+^ T cells in the periphery of aged mice and patients with pneumonitis, but not young mice or irAE-free patients following anti–PD-(L)1 therapy, suggest their predictive value for later incidence of lung toxicity. Elucidating such age-associated factors that exacerbate anti–PD-(L)1 therapy–induced irAE pathogenesis will also help to accommodate the need for a better therapeutic approach for irAEs.

The association between irAE development and ICOS^+^CD4^+^ T cells was forecasted in a previous study demonstrating that increased frequency of peripheral ICOS^+^CD4^+^ T cells was ameliorated by steroid treatment in patients with immune-related hepatitis and encephalomyelitis (37). However, it had not been clarified whether ICOS served as a functional prerequisite for irAE incidence and their kinetics before irAE onset had a predictive value. While PD-(L)1 blockade induced ICOS upregulation in pathogenic CD4^+^ T cells, age-associated PD-1 expression was also augmented by ICOS signaling. These reciprocal regulations confer a pivotal role of ICOS^+^CD4^+^ T cells in exacerbation of anti–PD-(L)1 therapy–related lung pathogenesis. Among the diverse effects of ICOS on T cells, ICOS signaling in CD4^+^ T cells may intrinsically promote their survival/accumulation in the lungs through activation of the PI3K/Akt pathway (28, 38), because disruption of the ICOS-ICOSL interaction reduced the frequency of not only lung-infiltrating B cells but also CD4^+^ T cells under an aged environment. Mechanistically, since Bcl-6 functions as a critical regulator of IL-21 production (31), it is conceivable that ICOS-mediated Bcl-6 induction may be a primary trigger for IL-21 production from CD4^+^ T cells. The fact that local IL-21 administration reverted the mitigation of lung pathogenesis induced by ICOSL blockade during anti–PD-(L)1 therapy suggests that ICOS-mediated local IL-21 production from aged CD4^+^ T cells is the most likely dominant driving force of anti–PD-(L)1 therapy–induced lung pathogenicity. Supporting this, augmented differentiation into auto-Ab–secreting cells and subsequent lung pathogenicity were dramatically ameliorated by IL-21 deficiency in anti–PD-(L)1 therapy–treated aged mice.

Considering their ability to produce IL-21 and to aid in B cell differentiation without expression of the classical Tfh marker CXCR5, the ICOS^+^PD-1^+^ effector CD4^+^ T cells enriched in the inflamed lung of aged mice resemble the recently reported human CXCL13^+^CXCR5^–^ age-associated helper T (ThA) cells that were found to accumulate during aging and in patients with systemic lupus erythematosus (39). Despite their similar B cell helper activity, irAE-inducing lung ICOS^+^CD4^+^ T cells did not prominently express CXCL13 or cytotoxicity-related factors such as *GzmK* or *Pfn1*, which are the characteristics of ThA cells. On the other hand, the lung-infiltrating ICOS^+^CD4^+^ T cells have a similar feature of T extrafollicular helper cells (34, 38) or osteopontin-producing (*Spp1*-producing) CD153^+^ senescence-associated T cells (32), both of which have been characterized by the expression of PD-1 and IL-21 but no CXCR5 expression. Our lung injury model, which is accompanied by surfactant dysfunction, increased alveolar size, and higher expression of COPD-associated gene signatures in the lung of anti–PD-1 therapy–treated aged mice, suggests that anti–PD-(L)1 therapy exacerbates the prelesioned state of lung damage. However, the detailed pathophysiology and target cells of the autoimmune response also remain to be elucidated. Moreover, since tissue residency and memory function of CD4^+^ T cells have also gained increasing attention in local and durable irAEs (40), further studies regarding the cellular ontogeny and persistency of age-associated autoreactive ICOS^+^CD4^+^ T cells and their interplay with B cells within the lung microenvironment are warranted to clarify the underlying mechanisms in irAE pathogenesis.

In addition to the pathogenic aged CD4^+^ T cells, age-associated environmental factors such as cognate self-antigen stimulation and inflammatory milieu in aged lung may be triggers or accelerators for local activation of self-reactive T and B cells, and subsequent anti–PD-(L)1 therapy–induced lung damage. This idea was supported by the transfer experiments using TCR-deficient mice, which revealed that irAE-like symptom did not develop under the young environment that did not supply the cognate stimulation for aged pathogenic CD4^+^ T cells and subsequent auto-Ab response. CXCL13 is also a potential age–associated environmental factor responsible for irAE development and dictates the site of irAE response through regulating local formation of TLS, especially in the aged environment (22, 26). Combined treatment with anti–PD-1 and anti-ICOSL Abs did not alter local CXCL13 levels in the lungs of tumor-bearing aged mice. From these phenomena, we hypothesized that (a) CXCL13-mediated initiation of local immune response and (b) subsequent ICOS-mediated mutual activation between CD4^+^ T cells and B cells at the local tissue independently contribute to the lung pathogenesis, but one of those responses alone may not be sufficient for irAE development. This notion is consistent with the fact that among ICI-treated patients, we found no correlation between the levels of ICOS^+^CD4^+^ T cells and CXCL13, despite their associated with irAE development. Therefore, it is quite reasonable that a combined assessment of these 2 risk factors would more accurately identify patients with irAEs, as demonstrated by our current pilot study.

CXCL13 may be a candidate target for irAE management (22, 25). However, increased levels of CXCL13 in tumor tissues have been suggested to be associated with a better response to anti–PD-(L)1 therapy (41), which may limit the utility of the CXCL13-targeting strategy as an option for irAE management, because of the risk of attenuating antitumor immunity. In this regard, targeting the ICOS signal that served as a “second hit” for irAE development may be an alternative management strategy that maintains CXCL13 function. To achieve this strategy, the impact of ICOS^+^CD4^+^ T cells on antitumor immune response should be well considered for optimized outcomes, because ICOS^+^ effector CD4^+^ T cells are also potentially associated with antitumor effects (42, 43). Our current study demonstrated that ICOSL blockade mitigated lung toxicity, but showed little association between ICO upregulation and antitumor effects in both aged mice and patients with cancer. These observations imply that early activation of ICOS^+^CD4^+^ T cells at the initial phase of ICI treatment may present an on-treatment window of opportunity for irAE prediction and be more strongly associated with irAEs than with antitumor immunity. A plausible explanation is that such a selective effect of ICOS signaling on irAE development or antitumor immunity depends on which cell types express ICOS in both organs with irAEs and tumor tissues, because ICOS-ICOSL interactions play a dual role in immune responses by acting on both effector T cells to promote their activation and Tregs to maintain their immunosuppressive activity (44). This may also explain why some clinical trials for the combined treatment with anti-ICOS agonistic Abs experienced a beneficial antitumor effect in anti–CTLA-4 therapy (45), but not in anti–PD-(L)1 therapy across several types of solid cancers (46, 47), despite an increased incidence of several types of irAE. Given that different types of cancer or self-organs have different Treg/effector T cell ratios, it is highly likely that the effects of agonistic ICOS stimulation on Tregs also vary by type of cancer or self-organ (48, 49). Based on these interpretations, it is necessary to determine the dependency of ICOS on both irAE pathology and antitumor immunity, especially in terms of the infiltration of Tregs and CD4^+^ T cells into tumor sites and tissues with irAEs, and their ICOS expression.

Because our current cohort was retrospective and consisted of a small number of patients, these limitations might lead to a bias in which mild toxicities such as dermatologic and gastrointestinal disorders were overlooked unless patients complained. On the other hand, pneumonitis and kidney failure were found with high frequency in our cohorts, as previously reported in other retrospective studies (33, 50, 51). Although these disease types did not fall into the most common spectrum of irAEs in the cohorts with other cancers besides NSCLC (50), the fact that many patients received a combination of ICI and chemotherapy in our NSCLC cohort might cause higher incidences of pneumonitis and hematologic toxicities (51). Because the current cohort was small, unlike in the Spearman’s rank correlation coefficient analysis, neither the change in ICOS^+^CD4^+^ T cells nor in CXCL13 was identified as an independent risk factor for irAEs (or pneumonitis) in the multivariate analysis. Therefore, further investigation with large-scale cohort studies across the diverse types of irAEs, cancers, and regimens will be necessary to validate the importance of ICOS^+^CD4^+^ T cells and CXCL13 in irAE development. In conclusion, the insight from our study into the relevance of ICOS^+^CD4^+^ T cells is potentially meaningful for a better understanding of irAE pathogenesis beyond lung toxicity, including skin disorders. However, it is worth noting that the ICOS/IL-21/auto-Ab–mediated organ toxicity is not necessarily applicable to all irAE pathologies, such as colitis (52). Therefore, it is necessary to classify irAEs from several immunological perspectives.

## Methods

### Sex as a biological variable.

Essentially the same data can be obtained from both male and female mice. However, since aged male mice exhibited more prominent phenotypes, males were utilized in the subsequent experiments. In the human cohort, sex was considered as a biological variable in both study design and analyses, and sex-based differences were assessed where appropriate.

### Mice.

Two-month-old young male WT C57BL/6J and BALB/c mice were purchased from Japan SLC, Inc. and CLEA Japan, respectively. TCRβ-deficient B6.129P2-*Tcrb^tm1Mom^*/J mice and IL-21–deficient mice were obtained from The Jackson Laboratory and Lexicon, respectively. They were back-crossed with C57BL/6J mice and maintained for 16–24 months for use as aged mice at the animal facility of Kyoto University. Young or aged TCRβ-deficient mice were reconstituted by adoptive transfer of 1 × 10^6^ CD4^+^ T cells that were isolated from the lungs of aged mice using Lympholyte-M (Cedarlane Laboratories) and EasySep Release Mouse Positive Selection Kit (STEMCELL Technologies).

### Tumor inoculation and Ab treatment.

MC38 (Kerafast) or CT26 (ATCC) cells were inoculated subcutaneously (4 × 10^5^) in C57BL/6J or BALB/c mice, respectively. Tumor growth was monitored by measuring the 2 largest perpendicular axes every 4 or 5 days until the tumor size (length × width) exceeded 400 mm^2^, and the size was expressed as tumor index, which is the square root of (length × width) (13). Two hundred micrograms of anti–PD-1 Ab (clone 29F.1A12, BioXCell), anti–PD-L1 Ab (clone 10F.9G2, BioXCell), anti-ICOSL Ab (clone UC10-4F10-11, BioXCell), and/or control rat IgG2a or IgG2b Ab (BioXCell) were injected intraperitoneally 4, 8, 12, and 16 days after tumor inoculation. In some experiments, 20 ng of recombinant mouse IL-21 (R&D Systems) was administered intranasally under anesthesia 1 day after Ab injection. For CD4^+^ T cell depletion, 200 μg of anti-CD4 Ab (clone GK1.5, BioXCell) was injected 10 days before and 3 days after tumor inoculation. For B cell depletion, a mixture of 150 μg of anti-CD19 Ab (clone 1D3, BioXCell), anti-CD22 Ab (clone Cy34.1, BioXCell), and anti-B220 Ab (clone RA3.3A1, BioXCell) was intraperitoneally injected 1 day before and 7 days after tumor inoculation. Two days after each injection, anti–rat Ig κ Ab (clone MAR 18.5, BioXCell) was administered (53).

### Measurement of cytokines and serological clinical markers.

Serum or BALF was harvested 15 days after tumor inoculation, unless mentioned otherwise. To harvest BALF, 500 μL of PBS was instilled into the lungs and allowed to equilibrate for 30 seconds before recollection and then centrifuged at 800*g* at 4°C for 5 minutes to isolate the cell and liquid components. The concentrations of murine SP-D, CXCL13, and IL-21, or human plasma CXCL13 and IL-6 were quantified by ELISA (R&D Systems). Other serological clinical markers were quantified using the JCA-BM6050 high-throughput chemical analyzer (Japan Electron Optics Laboratories).

### Flow cytometric analysis.

To discriminate blood-circulating immune cells from lung-infiltrating cells, APC-Cy7–labled anti-CD45 Ab (clone 30-F11, BioLegend; 3 μg/mouse) was intravenously injected into the mice 3 minutes before harvesting the tissues. Tissues were harvested, minced with razors, and analyzed for mRNA expression or digested with 2.5 mg/mL collagenase D (Roche) and 10 μg/mL DNase I (Sigma-Aldrich) for 30 minutes. Tissue-infiltrating or blood-circulating PBMCs were stained with Abs against the following proteins: mouse CD4 (clone GK1.5), CD8 (clone 53-6.7), B220 (clone RA3-6B2), CD19 (clone 6D5), CXCR5 (clone L138D7), ICOS (clone 7E.17G9), PD-1 (clone 29F.1A12), GL7 (clone GL7), CD95 (clone SA367H8), CD153 (clone RM153), IgD (clone 11-26c2a), IgG (clone poly4053) (all BioLegend), or anti-TCRβ (clone H57-597, Cytek) for mouse samples. In the analysis of patient samples, Abs against human CD3 (clone UCHT1), CD4 (clone RPA-T4), CD8 (clone RPA-T8), ICOS (clone C398.4A), CXCR5 (clone J252D4), CCR5 (clone J418F1), or CX3CR1 (clone 2A9-1) (all BioLegend) was used. For staining of intracellular cytokines, cells were stimulated with PMA/ionomycin in the presence of brefeldin A (all Sigma-Aldrich), and then stained with anti–IL-17A Ab (clone TC11-18H10.1, BioLegend) or IL-21R–Fc chimeric protein (R&D Systems), or anti–Bcl-6 (clone IG191E/A8, BioLegend), or anti-Foxp3 (clone MF23) Abs (BD Bioscience) using BD Cytofix/Cytoperm Buffer or Transcription Factor Buffer (BD Biosciences) (13). Multicolor immunofluorescence images were analyzed using a Fortessa (BD Biosciences). The data were analyzed using FlowJo software (Tree Star).

### RNA extraction, real-time quantitative PCR, and scRNA-seq.

Total RNA was extracted using TRI-Reagent (Molecular Research Center) and then were purified using an RNeasy Mini Kit (Qiagen). cDNA was synthesized through reverse transcription using ReverTra Ace (TOYOBO). Real-time quantitative PCR was run on a One-Step Real-Time PCR System (Applied Biosystems) using the probes specific for mouse *Ifng* (Mm.PT.58.41769240), *Il4* (Mm.PT.58.7882098), *Il21* (Mm.PT.58.7853071), *Cxcl13* (Mm.PT.58.31389616), *Cxcr5* (Mm.PT.58.45859964), *Pdcd1* (Mm.PT.58.29141957), *Rora* (Mm.PT.58.32675621), *Maf* (Mm.PT.58.42145949.g)*, Bcl6* (Mm.PT.58.32669842), and *Actb* (Mm.PT.39a.22214843.g) (all from IDT). Alternatively, SYBR qPCR Mix (TOYOBO) and the primers listed in Supplemental Table 6 were used. Expression levels for each gene were normalized to expression of *Gapdh* or *Actb* using the comparative 2^–ΔΔCt^ method.

scRNA-seq was conducted after enrichment of variable CD45^+^ immune cells from PBS-perfused whole lung tissues of pooled young and aged mice through density gradient centrifugation with Lympholyte-M, EasySep Release Mouse Biotin Positive Selection Kit (STEMCELL Technologies), and Dead Cell Remover (Miltenyi Biotec). Single-cell 3′ RNA-seq libraries were prepared using the Chromium Controller and Chromium Single Cell v3 Reagent Kit (10× Genomics). Libraries were qualified using an Agilent 2100, and then sequenced on a NovaSeq 6000 (Illumina). Initial data processing (demultiplexing, genome alignment, barcode counting, and UMI counting from raw sequencing data) was performed using Cell Ranger v7 (10× Genomics), and reads were mapped to the mm10 version of the mouse genome. Downstream analysis (quality control, normalization, dimensional reduction, clustering, and visualization of the raw count matrix) was performed using the Seurat package (v.4.3.0, https://satijalab.org/seurat/). Finally, 8666 and 6034 single cells from young and aged mice, respectively, were further analyzed. Principal component analysis was performed to reduce the number of feature dimensions, and the first 20 principal components with a resolution of 0.5 were adopted to identify clusters. Dimensional reduction was performed via uniform manifold approximation and projection (UMAP). Clusters of CD4^+^ T cell lineages were extracted, and dimensional reduction and reclustering were performed to identify differential clusters.

RNA-seq analysis with RNAs extracted from whole lung tissues was previously conducted (22) and heatmaps to show the relative expression of selected genes were calculated through *z*-score normalizing for each medial TPM value among the samples (NCBI GEO GSE184000).

### Evaluation of lung function.

Mice were anesthetized via intraperitoneal injection of pentobarbital sodium (40 mg/kg; Somnopentyl, Kyoritsu) and xylazine (2.0 mg/kg; Seractal, Bayer), and then were tracheostomized and connected to a computer-controlled small-animal ventilator (flexiVent, SCIREQ). Pulmonary functional parameters were measured with mechanical ventilation set at 150 breaths/min with a tidal volume of 10 mL/kg on 100% inspired oxygen, and at a positive end-expiratory pressure of 3 cmH_2_O. For each parameter, a coefficient of determination of 0.95 was the lower limit for accepting a measurement. To evaluate the linear intercept length of alveoli, 8 fields were randomly selected per lobe of H&E-stained lung tissues, and test lines were randomly drawn on images except airway and vascular structures (29). The alveolar intercept lengths were measured along with the test line, and the means were calculated.

### Histological evaluation and IHC.

Murine tissues were fixed with 4% (w/v) paraformaldehyde and routinely embedded in paraffin wax, sectioned, and stained with H&E or subjected to IHC as described previously (22). In brief, sections were deparaffinized and treated with 0.3% H_2_O_2_. Following antigen retrieval using citrate buffer (pH 6) and a pressure cooker (DAKO) or a microwave, the sections were stained with goat anti–mouse IgG (1:1000 dilution; BioLegend, catalog 405301) or anti–mouse CD138 (1:500 dilution; BD Pharmingen, catalog 553712) followed by HRP-conjugated anti–goat Ig Ab, and visualized with DAB solution (Nichirei). The integrated intensity and proportion of stained cells or TLS aggregates in regions of interest in IHC images were semiquantitatively analyzed using the Multi-stack Module and Hybrid Cell Count Software with the BZ-X800 All-in-One fluorescence microscope (Keyence).

### In situ RNA hybridization.

Slide-mounted tissue sections were prepared for RNAscope probe (RNA) hybridization and deparaffinized using general methods, and incubated in H_2_O_2_ at room temperature for 10 minutes followed by 1× Target Retrieval reagent (Advanced Cell Diagnostics) at 98°C for 15 minutes. Then, the sections were baked at 67°C for 30 minutes upon completion of Target Retrieval. Slides were then incubated in RNAscope Protease Plus (Advanced Cell Diagnostics) at 40°C for 30 minutes. The target mRNA in the tissues was hybridized by incubating in RNAscope buffered Z probes for *Icos* (Advanced Cell Diagnostics, 552451), *Cd4* (Advanced Cell Diagnostics, 406841-C2), and *Cd19* (Advanced Cell Diagnostics, 314711-C3) at 40°C for 2 hours. The signal was amplified using RNAscope Multiplex Fluorescent Detection Reagents v2 (Advanced Cell Diagnostics).

### In vitro stimulation of T cells.

Blood-circulating cells in mice were labeled through intravenous injection of biotin-conjugated anti-CD45 Ab, and the lungs were harvested and digested with DNase I and collagenase D. After lymphocyte enrichment via density gradient separation using Lympholyte-M, anti-CD45 Ab–stained blood-circulating cells were removed using anti-biotin Ab-coated magnetic beads (Miltenyi Biotec). Lung-infiltrating CD4^+^ T cells were further isolated using CD4 microbeads (Miltenyi Biotec), and stimulated with anti-CD3/anti-CD28 Ab–coated beads (Thermo Fisher Scientific) with or without plate-coated anti-ICOS agonistic Ab (5 μg/mL; clone 398.4A, BioXCell) or anti-CD153 agonistic Ab (5 μg/mL; clone RM153, eBioscience) for 30 hours before RNA extraction.

### Patients and approval for human experiments.

Patients with advanced and metastatic NSCLC who were treated with anti–PD-(L)1 Abs combined or not with chemotherapy or anti–CTLA-4 Ab, and for whom pre-/on-treatment (4 weeks after initial administration) samples were available, were identified at Kyoto University Hospital, and patients 20 years of age or older were included in the study (NSCLC; *n* = 44–49). This cohort was based on unbiased and comprehensive patient registration, and included 2 patients with rheumatoid arthritis and 1 patient with psoriasis, all of whom were treated with steroids. The other patients did not have anamnesis of autoimmune diseases and their treatment history available. Ficoll-separated PBMCs and plasma from the blood were cryopreserved until analysis. Samples were collected from September 2020 to October 2022 and were utilized in accordance with the Declaration of Helsinki. All the study protocols were approval by the Institutional Review Boards of Kyoto University (protocol no. G1012). Informed consent for all study procedures was obtained from all patients. Patient information is described in Supplemental Table 1.

PFS and OS were defined as the time from the start of ICI treatment until disease progression determined based on imaging and/or clinical observation, according to the treating pulmonologists’ clinical judgment, or until death from any causes determined by clinical records, respectively. PFS and OS were assessed until December 2023 in patients for whom sequential blood samples were available. Overall response was scored according to RECIST 1.1 criteria. Utilizing Common Terminology Criteria for Adverse Events v5.0, irAE development and toxicities based on standard laboratory values and clinical examinations were followed up from the initial anti–PD-1 therapy to 12 months after the final administration or December 2023, whichever came first. Any combination therapy–associated AEs were included as irAEs, as long as the patients received anti–PD-(L)1 therapy.

### Statistics.

Statistical significance was determined using Wilcoxon’s signed-rank test or Mann-Whitney *U* test when comparing 2 experimental groups. Multiple groups were compared using 1-way analysis of variance (ANOVA) followed by Tukey-Kramer post hoc tests. In some experiments, differences were determined using the Kruskal-Wallis test. PFS, OS, and the cumulative incidence rate of irAEs were estimated using the Kaplan-Meier method and log-rank test. Univariate and multivariable Cox proportional hazards analyses were also performed to evaluate the impact of covariates on these outcomes. For human samples, the Shapiro-Wilk test was used to test the normal distribution of variables (changes in the frequencies of CD4^+^ T cell subsets, CXCL13, and IL-6). To assess correlations among immunological and clinical parameters, including patient age, smoking history, performance status, and PD-L1 expression, we used Spearman’s partial correlation test. To evaluate the usefulness of immunological factors as predictors of irAE development, we utilized univariate and multivariate ROC analyses. Factors with a 95% AUC of greater than 50% in the univariate analysis were included in the multivariate analysis, in which we verified correlations between 2 variables based on an influence of third variable. Differences in the Bray-Curtis distance between 2 factors were assessed using the Adonis test. Statistical analyses were performed using Prism 7 (GraphPad Software), JMP Pro v16.1.0 (SAS Institute), and R v.4.3.3 (The R Foundation for Statistical Computing). Differences were considered statistically significant at *P* values of less than 0.05.

### Study approval.

All animal experiments were approved by the Institutional Animal Committee of Kyoto University (MedKyo 22069, 23055, and 24084) and performed in accordance with accepted guidelines. The study protocols of patients cohort were approved by the Institutional Review Boards of Kyoto University (protocol no. G1012). Written informed consent was obtained from all patients.

### Data availability.

The scRNA-seq and total RNA-seq data described in this study have been deposited in the NCBI (GEO GSE267557 and GSE184000, respectively). All data generated or analyzed during this study are included in this article. Values for all graphs are provided in the supplemental Supporting Data Values file. Further inquiries can be directed to the corresponding author.

## Author contributions

MY and HT designed the experiments. MY, YK, YF, H Yano, and HT performed experiments and analyzes data. MY, KM, MS, and HT performed the preclinical analyses. TY and KI contributed to bioinformatic analysis. TY, KC, HO, HY, KI, TO, and TH contributed to collection of clinical samples. MY, KM, KC, HO, H Yoshida, DH, TT, and HT discussed the results and interpreted data. MY and HT prepared the manuscript. All authors commented on and approved the manuscript.

## Supplementary Material

Supplemental data

Supporting data values

## Version 1 04/08/2025

In-Press Preview

## Version 2 05/22/2025

Electronic publication

## References

[1] Yu JX (2020). Trends in clinical development for PD-1/PD-L1 inhibitors. Nat Rev Drug Discov.

[2] Suijkerbuijk KPM (2024). Clinical and translational attributes of immune-related adverse events. Nat Cancer.

[3] Zhou X (2021). Treatment-related adverse events of PD-1 and PD-L1 inhibitor-based combination therapies in clinical trials: a systematic review and meta-analysis. Lancet Oncol.

[4] Oshima Y (2018). EGFR-TKI-associated interstitial pneumonitis in nivolumab-treated patients with non-small cell lung cancer. JAMA Oncol.

[5] Da L (2019). Organ-specific immune-related adverse events associated with immune checkpoint inhibitor monotherapy versus combination therapy in cancer: a meta-analysis of randomized controlled trials. Front Pharmacol.

[6] Nebhan CA (2021). Clinical outcomes and toxic effects of single-agent immune checkpoint inhibitors among patients aged 80 years or older with cancer: a multicenter international cohort study. JAMA Oncol.

[7] Matsukane R (2023). Systematic surveillance of immune-related adverse events in clinical practice and impact of subsequent steroid medication on survival outcomes. Int J Clin Oncol.

[8] Ghanbar MI, Suresh K (2024). Pulmonary toxicity of immune checkpoint immunotherapy. J Clin Invest.

[9] Bai X (2021). Early use of high-dose glucocorticoid for the management of irAE is associated with poorer survival in patients with advanced melanoma treated with anti-PD-1 monotherapy. Clin Cancer Res.

[10] Arbour KC (2018). Impact of baseline steroids on efficacy of programmed cell death-1 and programmed death-ligand 1 blockade in patients with non-small-cell lung cancer. J Clin Oncol.

[11] Kendal WS (2008). Dying with cancer: the influence of age, comorbidity, and cancer site. Cancer.

[12] Mogilenko DA (2022). Immune ageing at single-cell resolution. Nat Rev Immunol.

[13] Tsukamoto H (2015). IL-6-mediated environmental conditioning of defective Th1 differentiation dampens antitumour immune responses in old age. Nat Commun.

[14] Voruganti T (2023). Association between age and survival trends in advanced non-small cell lung cancer after adoption of immunotherapy. JAMA Oncol.

[15] Yu L (2024). IgG is an aging factor that drives adipose tissue fibrosis and metabolic decline. Cell Metab.

[16] Ramos-Casals M (2004). Systemic autoimmune diseases in elderly patients: atypical presentation and association with neoplasia. Autoimmun Rev.

[17] Huang X (2021). Age-associated changes in adverse events arising from anti-PD-(L)1 therapy. Front Oncol.

[18] Baldini C (2020). Impact of aging on immune-related adverse events generated by anti-programmed death (ligand)PD-(L)1 therapies. Eur J Cancer.

[19] Socinski MA (2021). Durvalumab after concurrent chemoradiotherapy in elderly patients with unresectable stage III non-small-cell lung cancer (PACIFIC). Clin Lung Cancer.

[20] Wang DY (2018). Fatal toxic effects associated with immune checkpoint inhibitors: a systematic review and meta-analysis. JAMA Oncol.

[21] Dunn C (2017). Older cancer patients in cancer clinical trials are underrepresented. Systematic literature review of almost 5000 meta- and pooled analyses of phase III randomized trials of survival from breast, prostate and lung cancer. Cancer Epidemiol.

[22] Tsukamoto H (2022). Aging-associated and CD4 T-cell-dependent ectopic CXCL13 activation predisposes to anti-PD-1 therapy-induced adverse events. Proc Natl Acad Sci U S A.

[23] de Moel EC (2019). Autoantibody development under treatment with immune-checkpoint inhibitors. Cancer Immunol Res.

[24] Flores-Chávez A (2022). Using autoantibodies to diagnose systemic autoimmune diseases triggered by immune checkpoint inhibitors: a clinical perspective. Crit Rev Immunol.

[25] Singh S Tertiary lymphoid structure signatures are associated with immune checkpoint inhibitor related acute interstitial nephritis. JCI Insight.

[26] Miura Y (2023). Predictive value of CXCL10 for the occurrence of immune-related adverse events in patient with renal cell carcinoma. Microbiol Immunol.

[27] Nurieva RI (2008). Generation of T follicular helper cells is mediated by interleukin-21 but independent of T helper 1, 2, or 17 cell lineages. Immunity.

[28] Simpson TR (2010). Regulation of CD4 T cell activation and effector function by inducible costimulator (ICOS). Curr Opin Immunol.

[29] Knudsen L (2010). Assessment of air space size characteristics by intercept (chord) measurement: an accurate and efficient stereological approach. J Appl Physiol (1985).

[30] Stahlbaum D (2023). Abnormalities on baseline chest imaging are risk factors for immune checkpoint inhibitor associated pneumonitis. Respir Med.

[31] Nurieva RI (2009). Bcl6 mediates the development of T follicular helper cells. Science.

[32] Fukushima Y (2022). cis interaction of CD153 with TCR/CD3 is crucial for the pathogenic activation of senescence-associated T cells. Cell Rep.

[33] Shankar B (2020). Multisystem immune-related adverse events associated with immune checkpoint inhibitors for treatment of non-small cell lung cancer. JAMA Oncol.

[34] Rao DA (2017). Pathologically expanded peripheral T helper cell subset drives B cells in rheumatoid arthritis. Nature.

[35] Lin X (2021). Peripheral blood biomarkers for early diagnosis, severity, and prognosis of checkpoint inhibitor-related pneumonitis in patients with lung cancer. Front Oncol.

[36] Cook SL (2024). Immune checkpoint inhibitors in geriatric oncology. Curr Oncol Rep.

[37] Bjursten S (2021). Early rise in brain damage markers and high ICOS expression in CD4^+^ and CD8^+^ T cells during checkpoint inhibitor-induced encephalomyelitis. J Immunother Cancer.

[38] Odegard JM (2008). ICOS-dependent extrafollicular helper T cells elicit IgG production via IL-21 in systemic autoimmunity. J Exp Med.

[39] Goto M (2024). Age-associated CD4^+^ T cells with B cell-promoting functions are regulated by ZEB2 in autoimmunity. Sci Immunol.

[40] Lozano AX (2022). T cell characteristics associated with toxicity to immune checkpoint blockade in patients with melanoma. Nat Med.

[41] Cabrita R (2020). Tertiary lymphoid structures improve immunotherapy and survival in melanoma. Nature.

[42] Xiao Z (2020). ICOS Is an indicator of T-cell-mediated response to cancer immunotherapy. Cancer Res.

[43] Duhen R (2022). PD-1 and ICOS coexpression identifies tumor-reactive CD4^+^ T cells in human solid tumors. J Clin Invest.

[44] Martin-Orozco N (2010). Melanoma cells express ICOS ligand to promote the activation and expansion of T-regulatory cells. Cancer Res.

[45] Liakou CI (2008). CTLA-4 blockade increases IFNgamma-producing CD4+ICOShi cells to shift the ratio of effector to regulatory T cells in cancer patients. Proc Natl Acad Sci U S A.

[46] Yap TA (2022). First-in-human phase I/II ICONIC trial of the ICOS agonist vopratelimab alone and with nivolumab: ICOS-High CD4 T-cell populations and predictors of response. Clin Cancer Res.

[47] Hilton JF (2024). INDUCE-2: A phase I/II, open-label, two-part study of feladilimab in combination with tremelimumab in patients with advanced solid tumors. Cancer Immunol Immunother.

[48] Nagase H (2017). ICOS^+^ Foxp3^+^ TILs in gastric cancer are prognostic markers and effector regulatory T cells associated with Helicobacter pylori. Int J Cancer.

[49] Sainson RCA (2020). An antibody targeting ICOS increases intratumoral cytotoxic to regulatory T-cell ratio and induces tumor regression. Cancer Immunol Res.

[50] Gougis P (2024). Clinical spectrum and evolution of immune-checkpoint inhibitors toxicities over a decade-a worldwide perspective. EClinicalMedicine.

[51] Tanaka H (2024). Comparison of immune checkpoint inhibitor plus chemotherapy or ipilimumab plus nivolumab-based therapy for NSCLC patients with PD-L1 TPS (1-49 %): TOPGAN2023-01. Eur J Cancer.

[52] van Eijs MJM (2023). Highly multiplexed spatial analysis identifies tissue-resident memory T cells as drivers of ulcerative and immune checkpoint inhibitor colitis. iScience.

[53] Keren Z (2011). B-cell depletion reactivates B lymphopoiesis in the BM and rejuvenates the B lineage in aging. Blood.

