# Supplementary material for: ICOS+CD4+ T cells define a high susceptibility to anti–PD-1 therapy–induced lung pathogenesis
Source: JCI Insight. 2025 Apr 8;10(10):e186483. doi: 10.1172/jci.insight.186483 (PMC12128994; doi:10.1172/jci.insight.186483)
Supplement: Supplemental data [file jciinsight-10-186483-s137.pdf]

Supplemental materials for

ICOS+CD4 T cells define a high susceptibility to anti-PD-1 therapy-induced lung pathogenesis

Mari Yokoi, Kosaku Murakami, Tomonori Yaguchi, Kenji Chamoto, Hiroaki Ozasa, Hironori Yoshida, Mirei Shirakashi, Katsuhiro Ito, Yoshihiro Komohara, Yukio Fujiwara, Hiromu Yano, Tatsuya Ogimoto, Daiki Hira, Tomohiro Terada, Toyohiro Hirai, Hirotake Tsukamoto*

*Corresponding author: Hirotake Tsukamoto

Email: tsukamoto.hirotake.4j@kyoto-u.ac.jp

This PDF file includes:

Figures S1 to S10

Table S1 to S6

Supplementary Figures and Tables

Supplementary Figure 1

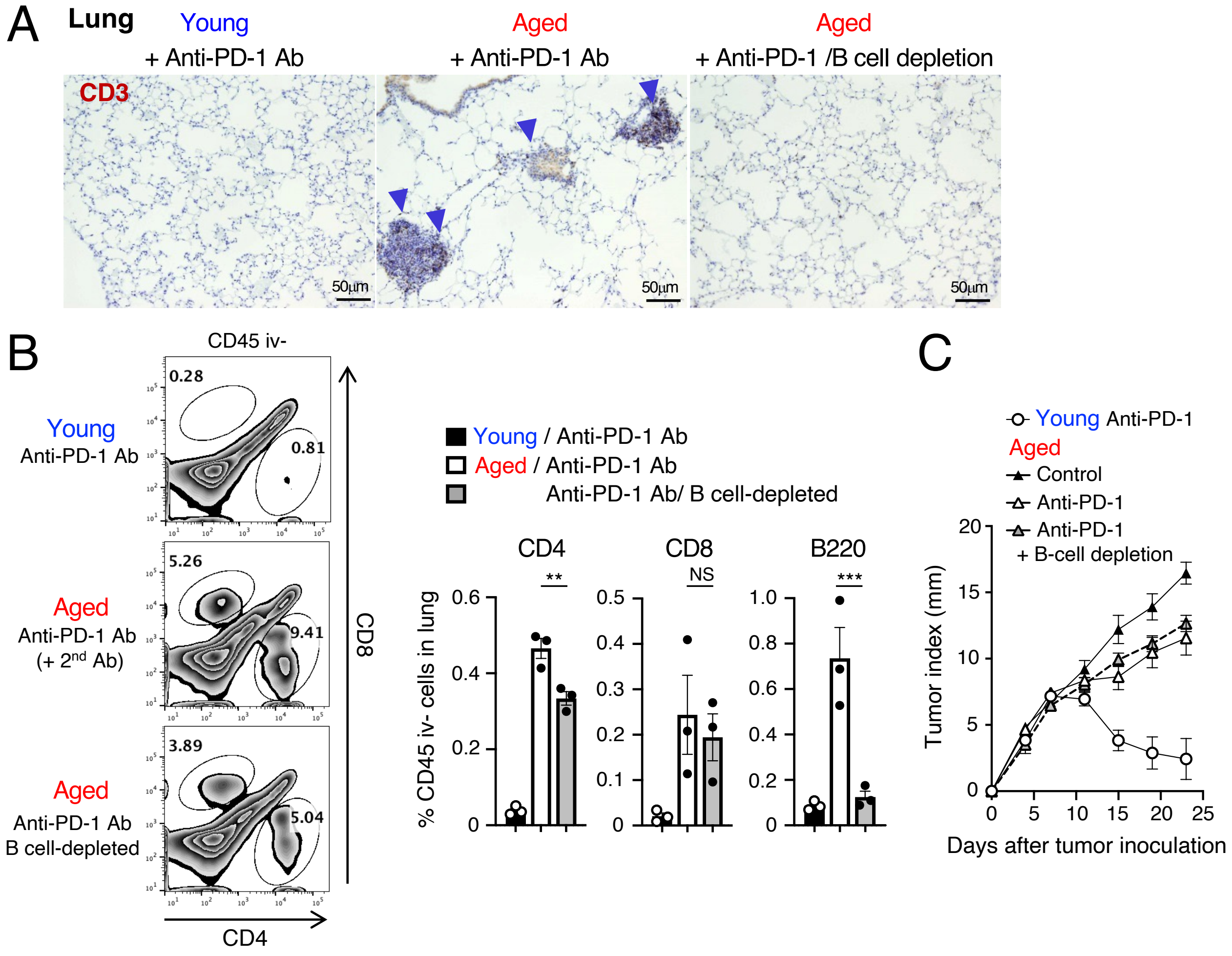

Fig. S1. The B-cell response is indispensable for anti-PD-1 therapy-induced lung pathogenesis but does not affect the anti-tumor effect. After B-cell depletion, anti-PD-1 Ab were injected in MC38-bearing aged mice. (**A**) Five days after the last treatment, lymphocytic aggregates in the lung were subjected to IHC stained with anti-CD3 Ab (brown). Scale bars indicate 50 µm. (**B**) Anti-CD45 Ab were intravenously (iv) injected before harvesting the lungs. Representative dot plots (left panels) and the frequencies (right graphs) of CD45 iv staining-negative lung-infiltrating T and B cells are shown (n = 5). (**C**) Outgrowth of MC38 in young or aged mice were monitored over time (n = 6) and tumor sizes were expressed as tumor index (square root (length x width)). Data are mean ± SEM of at least two independent experiments with similar results. $^{**}p < 0.01$, $^{***}p < 0.001$.

Supplementary Figure 2

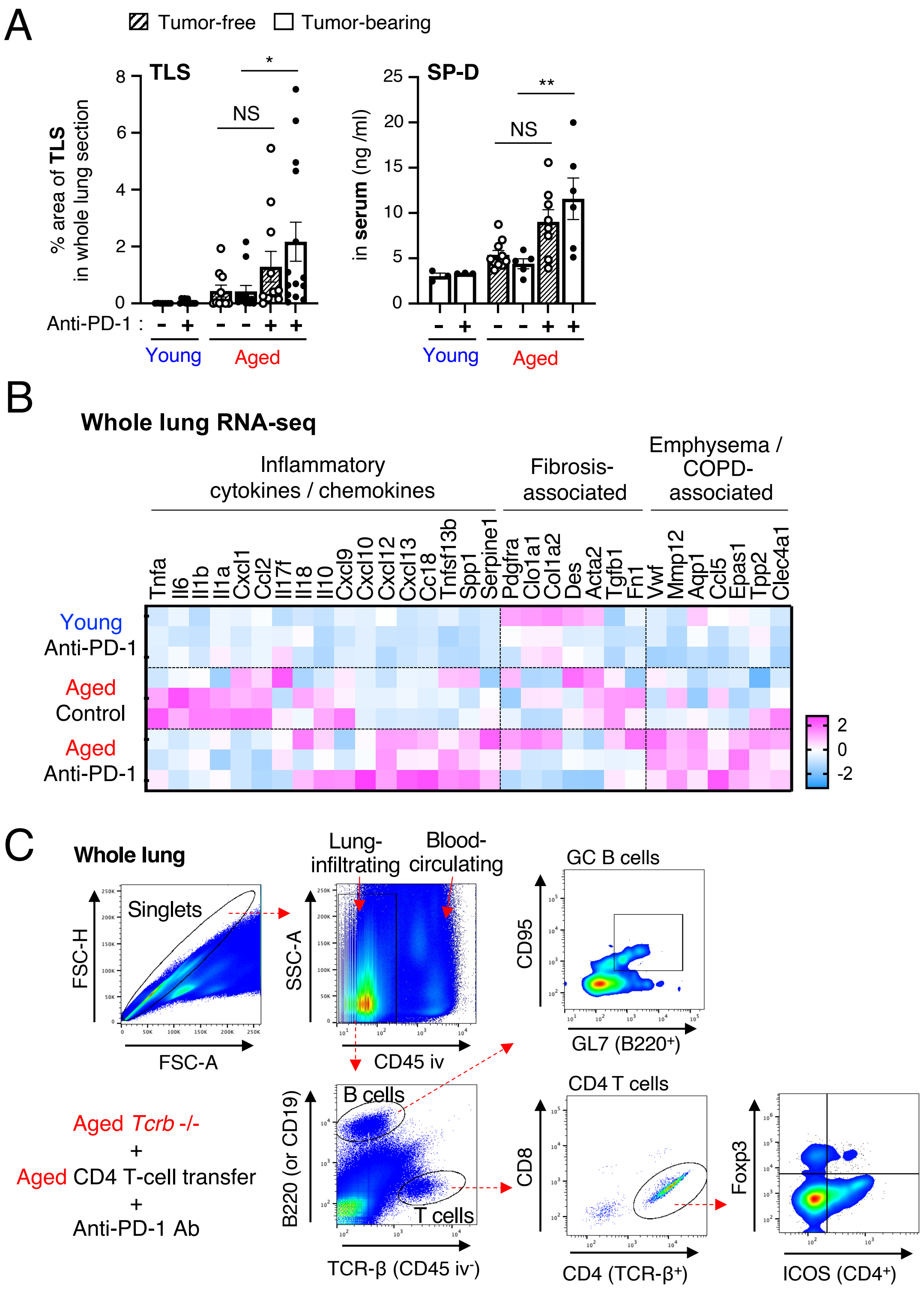

Fig. S2. Characterization of anti-PD-1 therapy-induced lung pathogenesis in tumor-bearing aged mice (**A**) Tumor-free or MC38-bearing mice were treated with control or anti-PD-1 Ab. Area of ectopic immune cell aggregates (TLS) in lung sections and serum SP-D levels were evaluated by H&E staining and ELISA,

respectively. Data are mean ± SEM of at least two independent experiments with similar results. $^{*}p < 0.05$, $^{**}p < 0.01$. (**B**) Heatmap shows the relative expression levels of inflammatory genes, fibrosis-associated genes, and emphysema/COPD-associated genes in total RNA sequencing analysis of whole lung tissues from tumor-bearing mice. (**C**) Aged WT CD4 T cells were transferred into aged TCRβ-deficient mice, and then anti-PD-1 Ab was injected. Gating strategy for lung-infiltrating B cells and T cells (defined by CD45 $iv^{-}B220^{+}$ or $CD19^{+}$, and CD45 $iv^{-}TCR\text{-}\beta^{+}CD4^{+}$ or $CD8^{+}$, respectively). GC B cells were defined by $B220^{+}CD95^{+}GL7^{+}$ cells.

Supplementary Figure 3

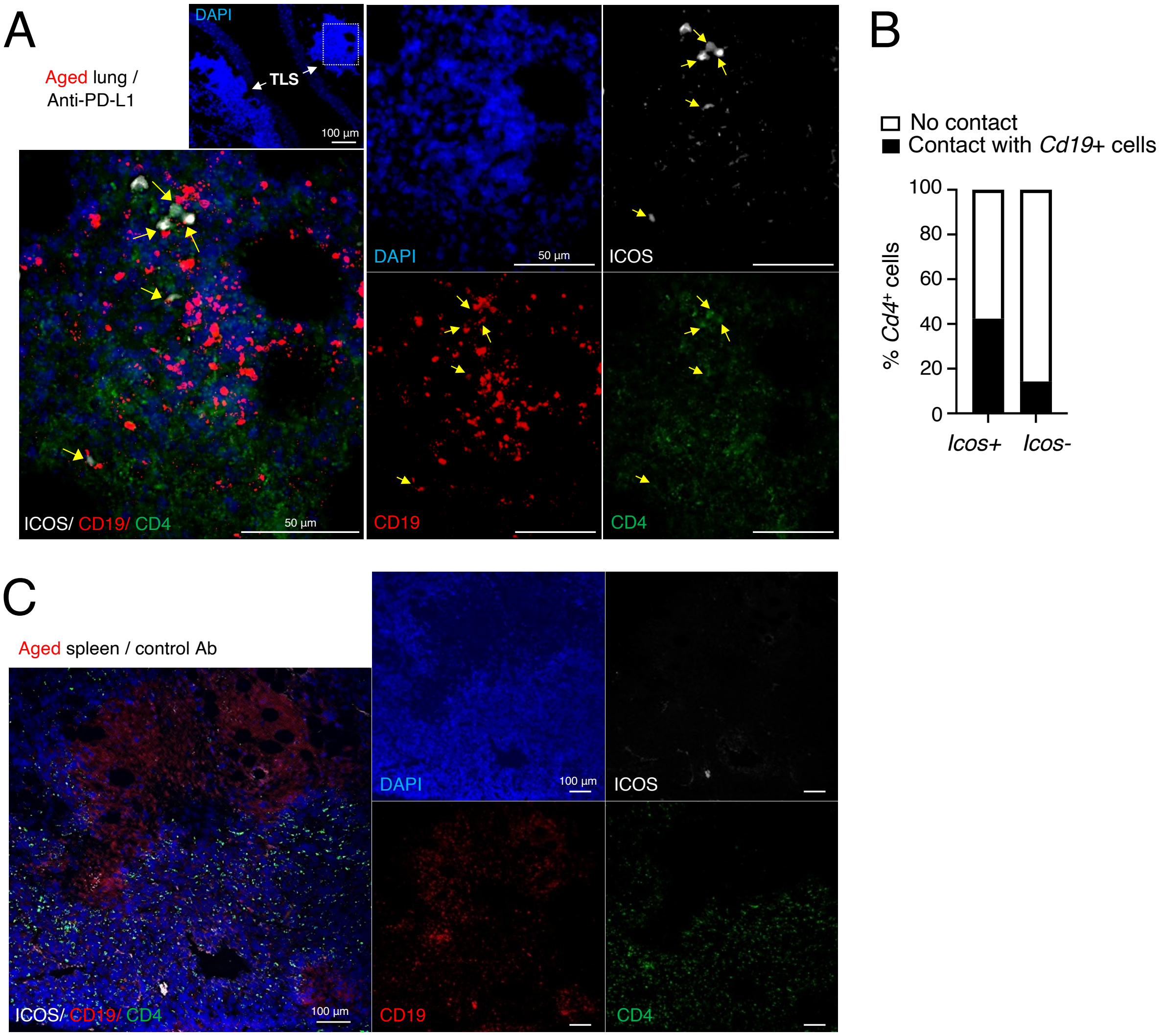

Fig. S3. Histological analysis of lymphocytic aggregation in lung from anti-PD-1 Ab-treated aged mice. MC38-bearing aged mice were treated with anti-PD-1 Ab (A and B) or control Ab (C) 3 times. Seven days after the last treatment, lungs (A and B) or spleen (C) were analyzed. (**A** and **C**) Representative images of DAPI staining, in-situ RNA hybridization (white: ICOS, red: CD19, green: CD4), and merged images of aged lung of spleen are shown. Spleen from control Ab-treated aged mice were utilized as a negative control for ICOS staining. Scale bars indicate 100 or 50 µm. Yellow arrows indicate the boundaries where *Icos*-expressing *Cd4*$^{+}$ cells interact with *Cd19*$^{+}$ cells. (**B**) Percentage of *Icos*$^{+}$*Cd4*$^{+}$ cells or *Icos*$^{-}$*Cd4*$^{+}$ cells that interacted with *Cd19*$^{+}$ cells within the TLS are indicated in black color. Each bar graph includes *Cd4*$^{+}$ cells that did not interact with *Cd19*$^{+}$ cells (white), and the total cells are shown as 100 % in each fraction.

Supplementary Figure 4

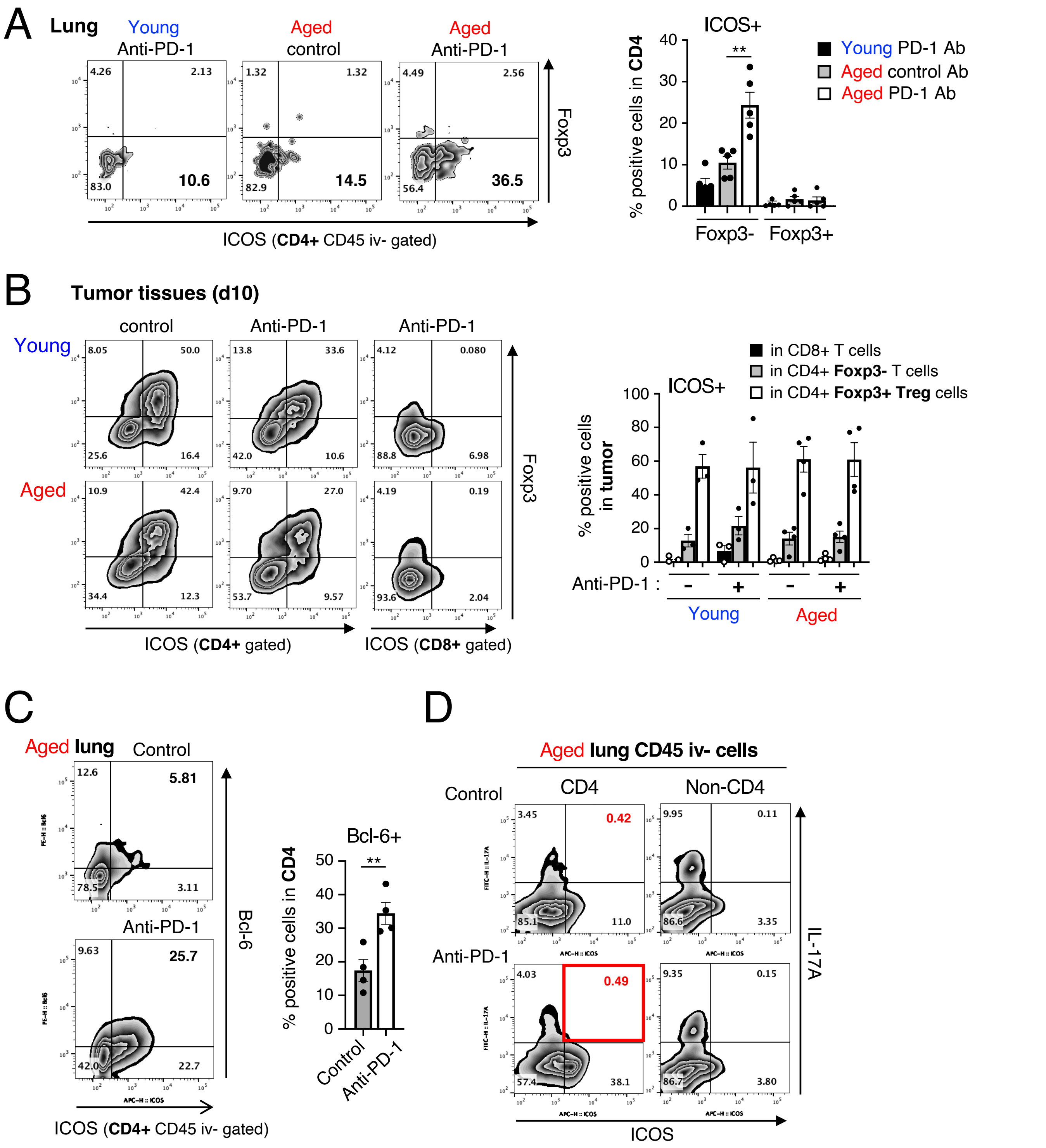

Fig. S4. Phenotypic characterization of ICOS^{+} CD4 T cells from tumor-bearing aged mice. MC38-bearing young or aged mice were treated with anti-PD-1 Ab 3 times. Two days after the last treatment, lung- or tumor-infiltrating ICOS^{+}CD4 T cells were analyzed. (**A**) Representative dot plots (upper panels) and the frequencies (lower graphs) of CD45 iv staining-negative lung-infiltrating Foxp3^{-} or Foxp3^{+} ICOS^{+}CD4 T cells (n = 5). (**B**)

Representative dot plots (left panels) and the frequencies (right graphs) of tumor-infiltrating $Foxp3^-$ or $Foxp3^+$ $ICOS^+$CD4 T cells (n = 3–4). (**C**) Representative dot plots of lung-infiltrating $Bcl6^+$ $ICOS^+$ cells (left panels) and the frequency of $Bcl\text{-}6^+$ cells in CD4 T cells (right graph) are shown. (**D**) IL-17-producing cells were analyzed by intracellular cytokine staining of lung CD4 T cells or CD4 negative cells from aged mice with or without anti-PD-1 Ab treatment. Data are representative of at least three independent experiments with similar results (mean ± SEM). ***p* < 0.01.

Supplementary Figure 5

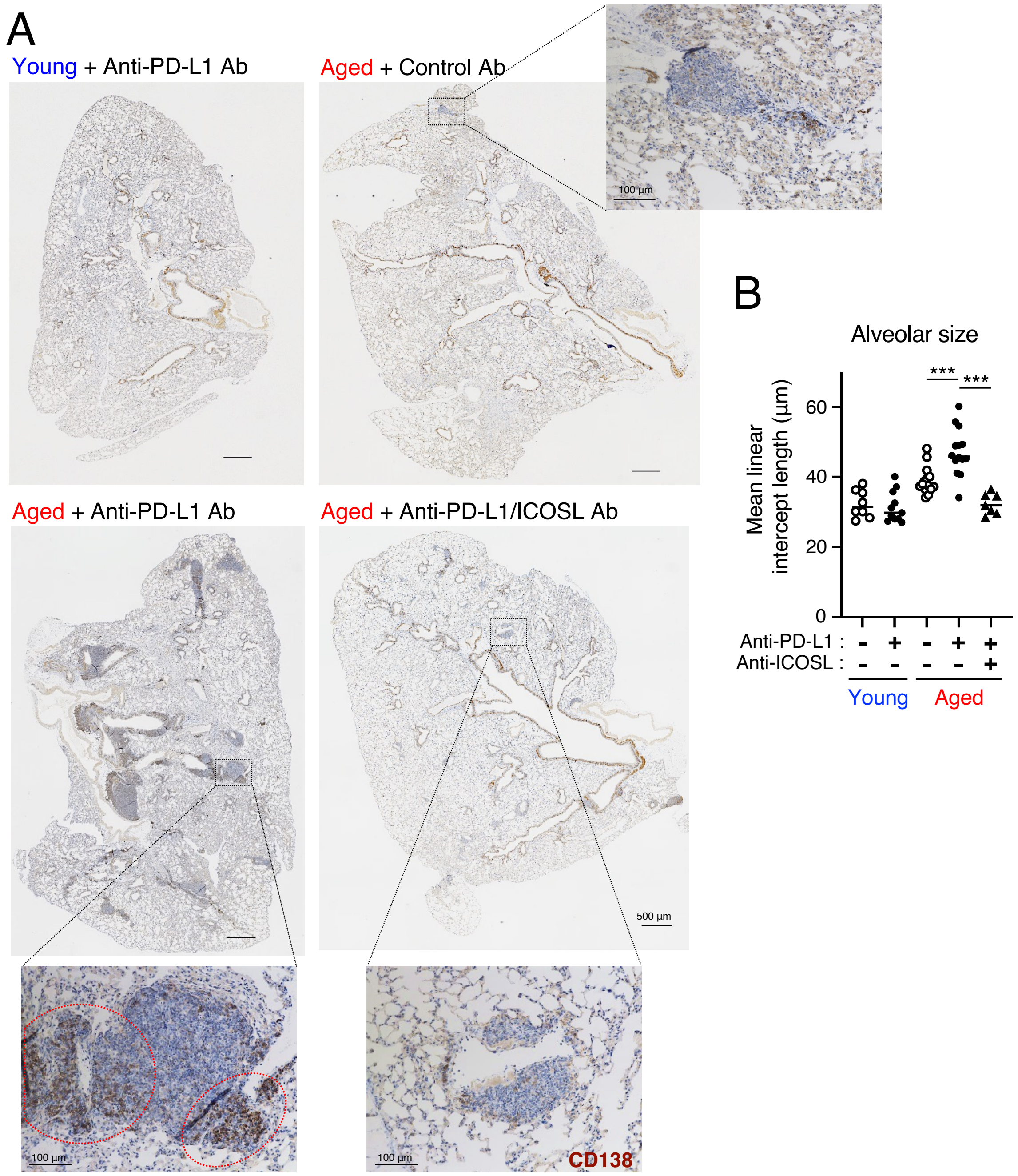

Fig. S5. Representative images of whole lung section with TLS and CD138⁺ cells. (**A** and **B**) MC38-bearing young or aged mice were treated with anti-PD-1 Ab in combination with or without anti-ICOSL. Ten days after the last treatment, lungs were harvested and stained with anti-CD138 Ab. Scale bars indicate 500 µm or 100 µm. The regions of interest (ROIs), including TLS and $CD138^+$ cells (brown), are enlarged in the other panels (A). For the measurement of mean linear intercept length of alveolar, eight fields were randomly selected per one lobe of H&E-stained lung tissues, and test lines were randomly drawn on images except airway and vascular structures. Calculated mean linear intercept lengths from indicated mice are shown (B). Values represent mean ± SEM of independent experiments. ***$p < 0.001$.

Supplementary Figure 6

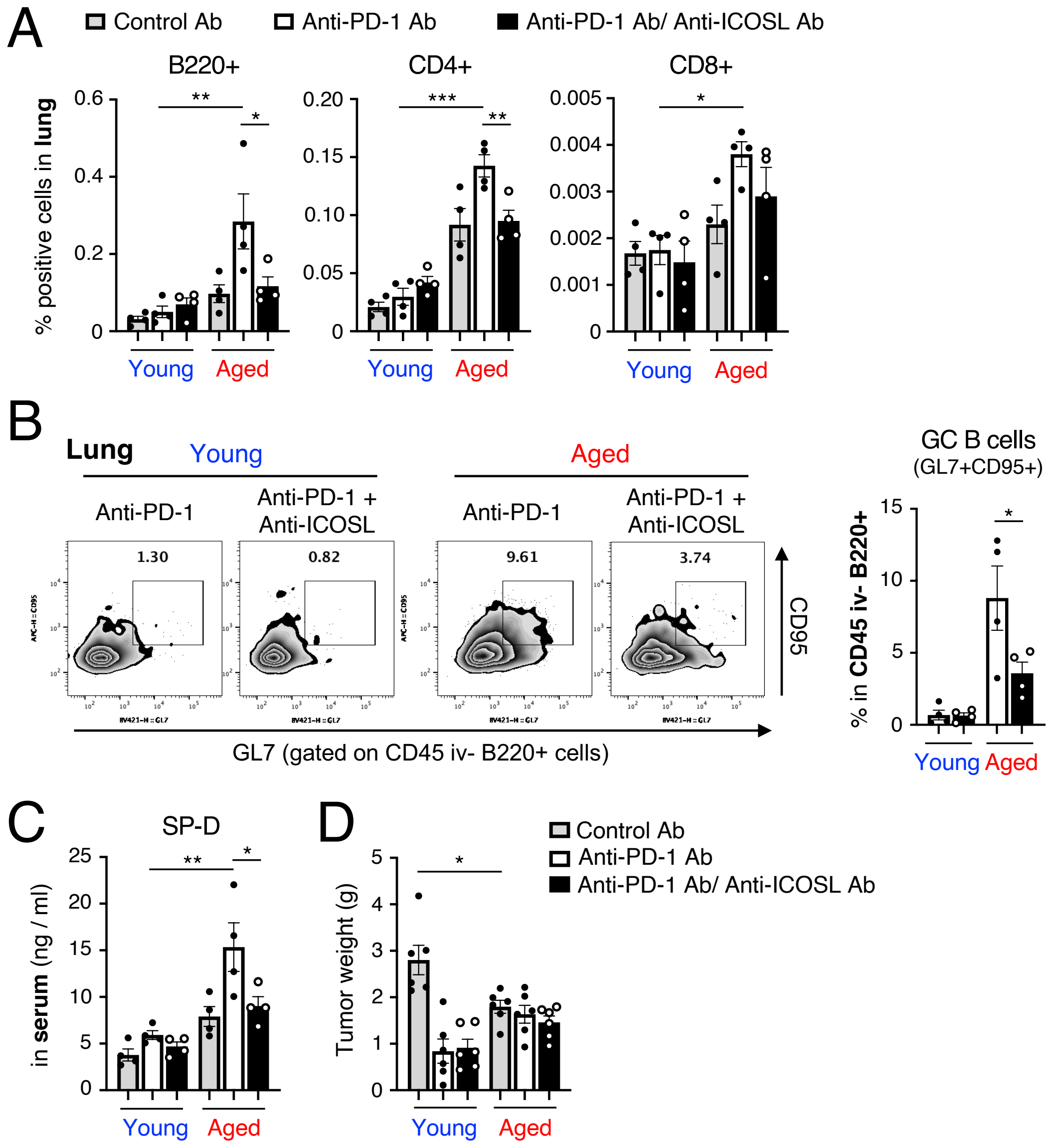

Fig. S6. Anti-PD-1 therapy-induced lung pathogenesis was attenuated by ICOSL blockade in aged mice. CT26-bearing young or aged Balb/c mice were treated with anti-PD-1 Ab combined with or without anti-ICOSL Ab. (**A** and **B**) Frequencies of CD45 iv staining-negative lung-infiltrating B cells and T cells (A), representative dot plots of GC B cells (B, left), and their frequency (B, right) are shown. (**C** and **D**) The levels of SP-D in the serum (C) and weight of tumor tissues (D) in indicated groups were measured 21 days after tumor inoculation. Data are mean ± SEM from two independent experiments with similar results. *p < 0.05, **p < 0.01, ***p < 0.001.

Supplementary Figure 7

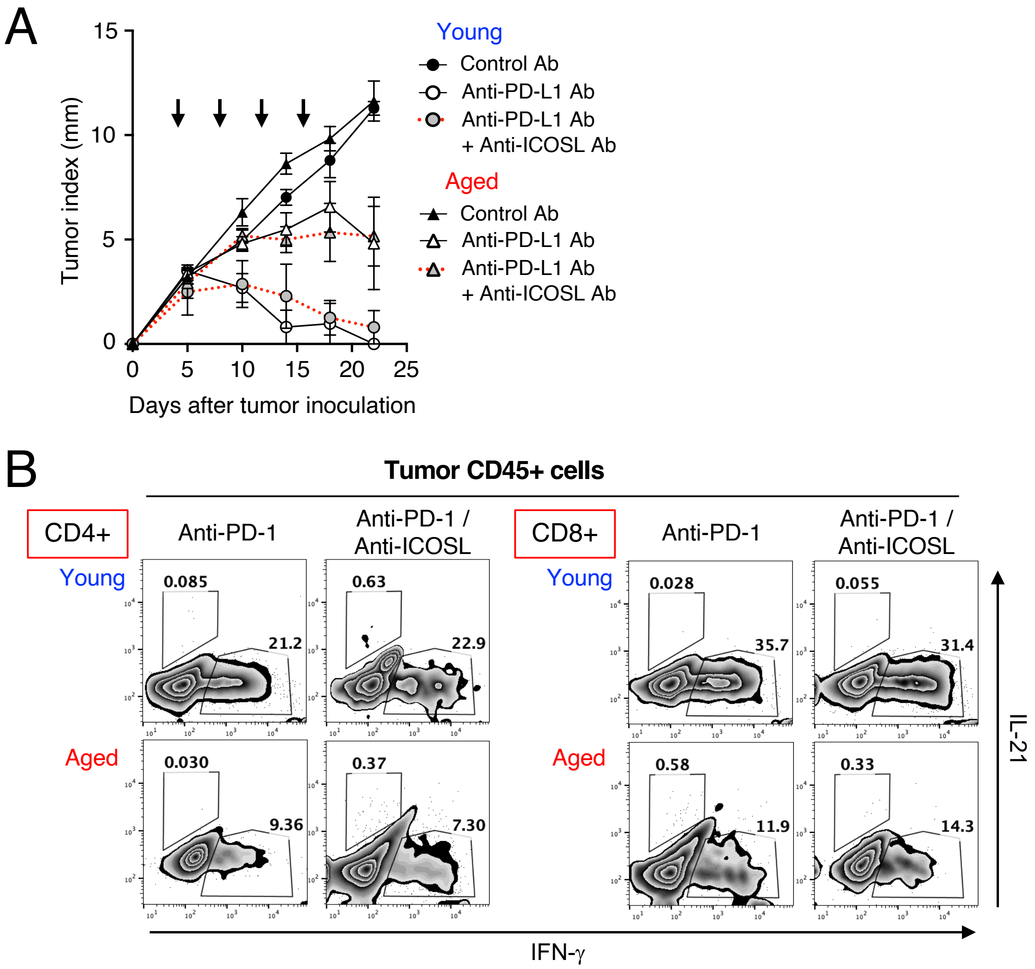

Fig. S7. ICOSL blockade does not impair anti-tumor effect of anti-PD-1 therapy. MC38-bearing young or aged mice were treated with anti-PD-L1 and anti-ICOSL Abs. (**A**) Tumor progression was monitored over time (n = 6), and tumor size was expressed as the tumor index (square root (length × width)). The data represent mean ± SEM. (**B**) Two days after the second treatment, tumor-infiltrating CD8 or CD4 T cells were analyzed for production of IFN-γ and IL-21 using intracellular cytokine staining. Data are representative of at least two independent experiments with similar results.

Supplementary Figure 8

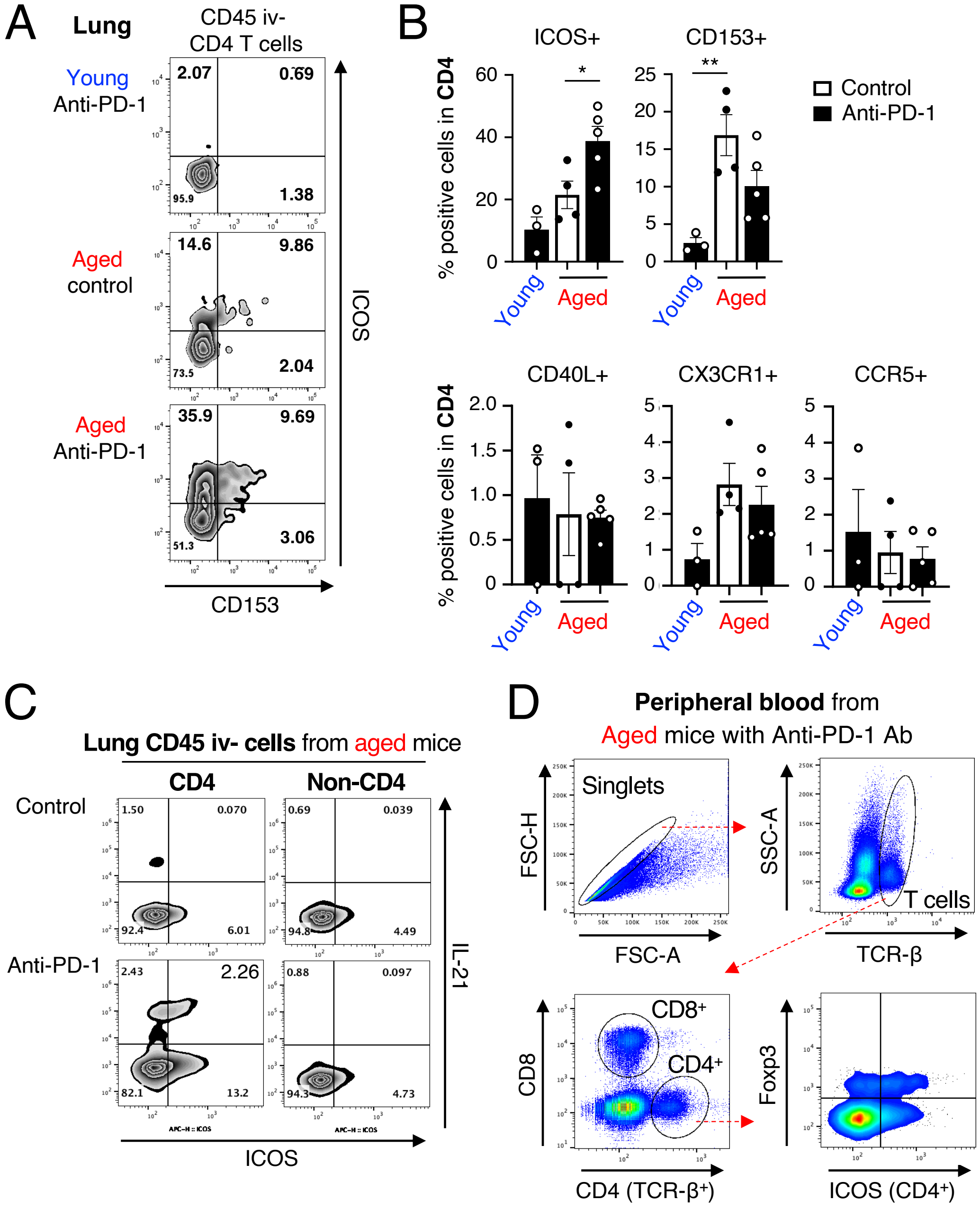

Fig. S8. Characterization of lung-infiltrating cells in aged mice. MC38-bearing young or aged mice were treated with anti-PD-1 Ab. (**A** and **B**) Representative plots showing the expression of ICOS and/or CD153 (A) and the frequencies of indicated molecule-expressing cells (B) in CD45 iv staining-negative lung-infiltrating CD4 T cells are shown (mean ± SEM; n = 3–5). *$p < 0.05$, **$p < 0.01$. (**C**) Lung-infiltrating CD4 T cells or CD4-negative cell populations were harvested from MC38-bearing aged mice 2 days after the last Ab treatment and were analyzed for IL-21-producing ability. Data are representative of two independent experiments with similar results. (**D**) Gating strategy for peripheral CD4 T cells in the blood and their ICOS expression.

Supplementary Figure 9

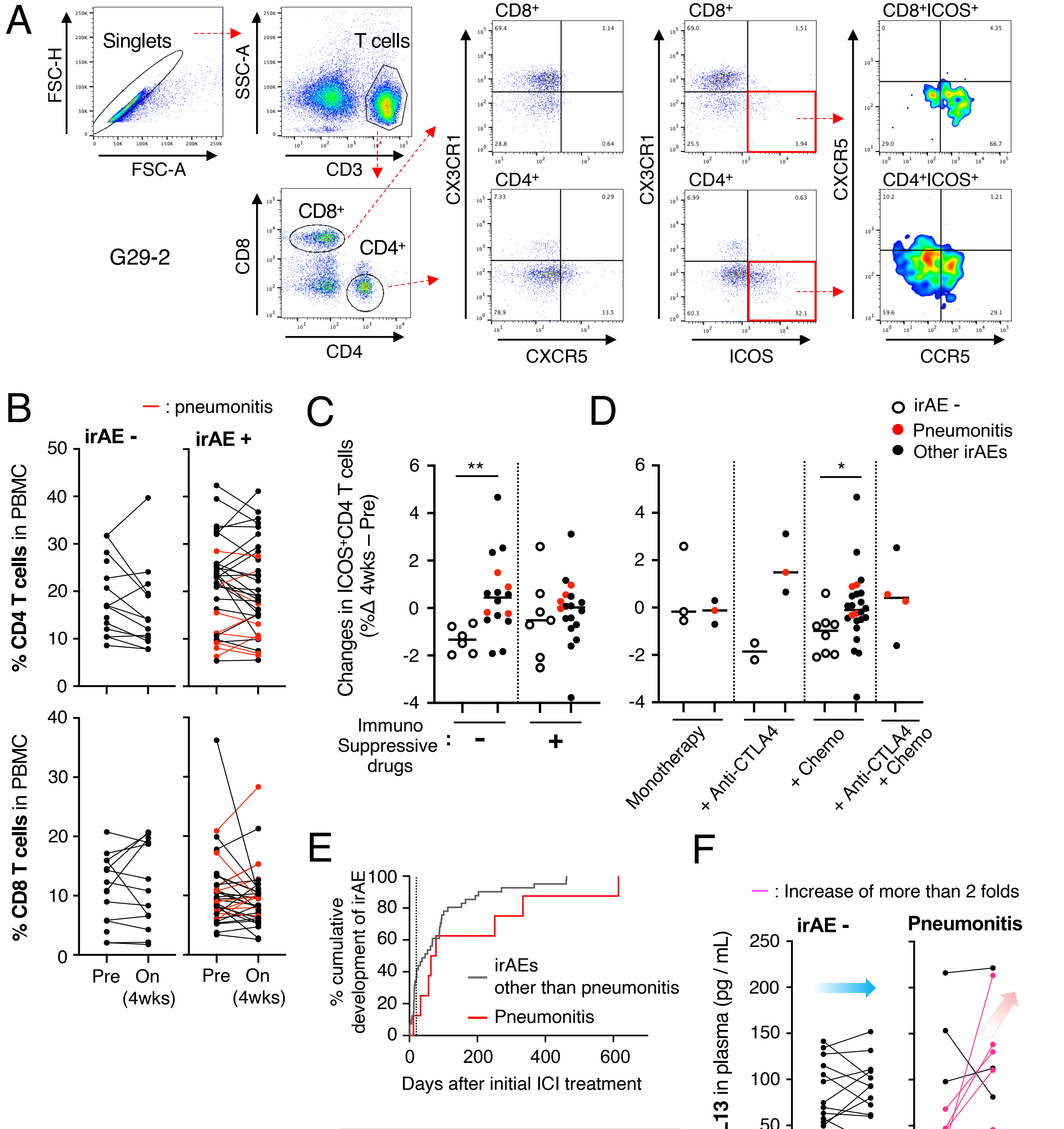

	n	Median (day)	CI
irAE	49	56.0	21-88
Pneumonitis	8	70.5	12-334
irAEs other than pneumonitis	41	46.0	16-88

Fig. S9. Analyses of peripheral T subsets in patients with NSCLC and irAE. (**A**) Gating strategy for ICOS+

T cells in PBMCs from patients with non-small cell lung carcinoma (NSCLC). (**B**) The frequencies of CD4 (upper panels) and CD8 (lower panels) T cells in PBMC were analyzed before (Pre) and 4 weeks after initial anti-PD-(L)1 therapy (On) in patients with irAE (n = 34) or without irAEs (n = 14). The red lines indicate the kinetics in the patients with pneumonitis. (**C** and **D**) Anti-PD-1 therapy-treated patients were stratified by the usage of immunosuppressive drugs (C), or the combination regimens (D), and their changes in the frequency of $ICOS^{+}$CD4 T cells in PBMCs are shown. The Mann-Whitney test was used for comparisons within each subgroup. *$p < 0.05$, **$p < 0.01$. (**E**) Kaplan Meier curve for the incidence of irAE or pneumonitis in patients with NSCLC treated with anti-PD-(L)1 therapy. (**F**) CXCL13 levels in plasma were analyzed before (Pre) and 4 weeks after initial anti–PD-(L)1 therapy (On) in patients with pneumonitis (n = 8) or without irAEs (n = 12). The red lines indicate the patients with > 2 folds increase of CXCL13 levels.

Supplementary Figure 10

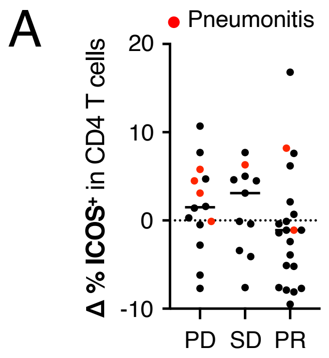

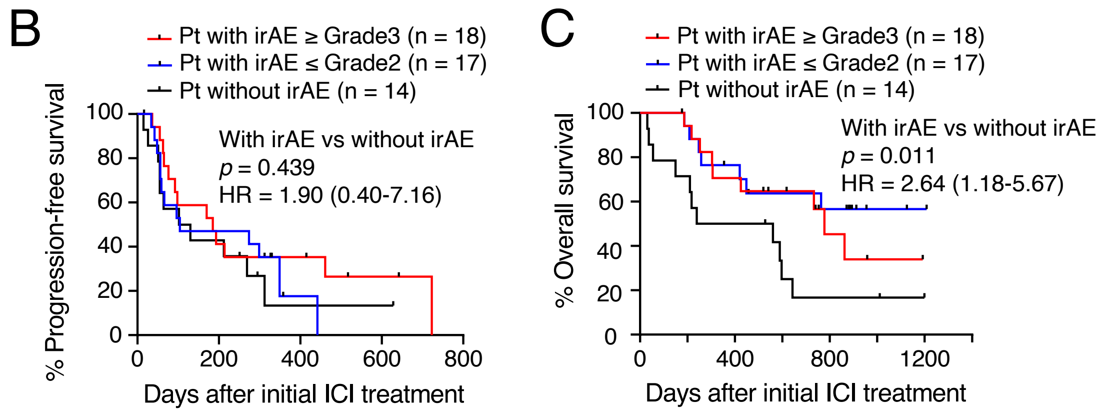

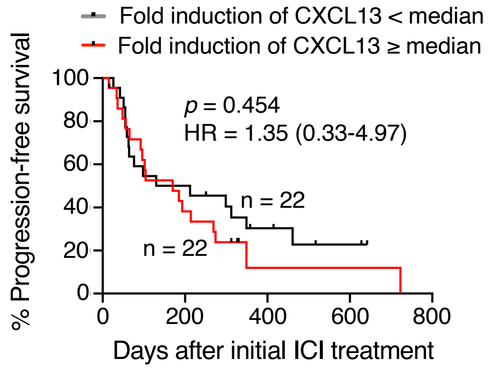

Multivariable Cox Proportional Hazards Analysis with Overall Survival

	HR	95% CI
Δ %ICOS+CD4+	0.87	0.63 - 1.18
Fold induction of CXCL13	1.24	0.72 - 1.83
Fold induction of IL-6	1.12	0.82 - 1.44
Age	0.98	0.94 - 1.03
irAE	2.25	0.83 - 5.98

	HR	95% CI
Fold induction of IL-6	1.12	0.88 - 1.48
Age	0.98	0.94 - 1.02
irAE	2.18	0.91 - 4.83

Fig. S10. Relationship between patient prognosis incidence of anti-PD-(L)1 therapy-induced irAEs. (**A**) Change in the ratio of ICOS$^+$CD4 T cells were plotted based on the best overall response. Red dots indicated patients with irAE pneumonitis. PD, progressive disease; SD, stable disease; PR, partial response. (**B** and **C**) Kaplan Meier curves of progression-free survival of NSCLC patients with (≥ Grade 3 or ≤ Grade 2) or without irAEs (B, upper panel), and with or without increased levels of CXCL13 during anti-PD-(L)1 therapy (B, lower panel). Kaplan Meier curves of overall survival of NSCLC patients with (≥ Grade 3 or ≤ Grade 2) or without irAEs (C, upper panel), and Multivariable Cox proportional hazards analysis for overall survival. The analysis includes %ICOS$^+$CD4 T cells, fold induction of CXCL13, fold induction of IL-6, age, and the presence of irAEs as covariates (C, lower panel). Due to multicollinearity among %ICOS$^+$CD4 T cells, fold induction of CXCL13, and irAEs, results excluding values of ICOS and CXCL13 are also presented in the lower. HR, Hazard ratio; CI, confidence interval.

Supplementary Table 1

Clinical characteristics of NSCLC patients who received anti-PD-1/PD-L1 therapy

		Non-irAE (n=14)	irAE (n=35)
Aged, mean (SD)		68.6 (11.7)	65.9 (10.9)
Sex, n (%)	Male	10 (26.3)	28 (73.7)
	Female	4 (36.4)	7 (63.6)
Smoking history, n (%)	Current or Former	12 (27.3)	32 (72.7)
	Never	2 (40.0)	3 (60.0)
PS, n (%)	0-2	11 (23.9)	35 (76.1)
	3-4	3 (100.0)	0 (0.0)
PD-L1 tumor proportion score	≥50%	2 (22.2)	7 (77.8)
	1-49%	5 (31.3)	11 (68.8)
	<1%	2 (18.2)	9 (81.8)
	unknown	5 (38.5)	8 (61.5)
ICI, n (%)	Anti-PD-1	4 (33.3)	8 (66.7)
	Anti-PD-L1	8 (28.6)	20 (71.4)
	Anti-PD-1+CTLA4	2 (22.2)	7 (77.8)
Chemotherapy (+ DEX) [a], n (%)	Yes	8 (22.2)	28 (77.8)
	No	6 (46.2)	7 (53.8)
Use of immunosuppressants (IS) [b], n (%)	Yes [c]	8 (29.6)	19 (70.3)
	No	6 (27.3)	16 (72.7)
irAE, n (%)	Hematologic		14
	Pneumonitis		8
	Nephritis		5
	Skin		5
	Thyroid		4
	Hepatitis		2
	Other [d]		10

DEX, dexamethasone; irAE, immune-related adverse event; IS, immunosuppressants; PS, performance status; PSL, prednisolone; SD, standard deviation. [a] All the patients who received ICIs combined with chemotherapy were treated with DEX as premedication. [b] IS include orally administered steroids (n=11), tacrolimus (n=1), inhaled steroids (n=3), as well as topical steroids. [c] IS-treated patients are defined as those who received IS in the period from one month before to one month after initial ICI administration. [d] Other includes the irAE of infusion reaction (n=3), fever (n=2), adrenal (n=1), cardiac (n=1), diarrhea (n=1), hypophysitis (n=1), neurologic (n=1).

Supplementary Table 2

Spearman's rank correlation matrix and the significance of relevant variables in the patients with NSCLC

	Age	Sex (male)	Smoking	PS	Chemotherapy	Use of IS	irAE	Pre % ICOS+ CD4+ T cells in PBMC	4wks % ICOS+ CD4+ T cells in PBMC	Δ % ICOS+ CD4+ T cells in PBMC	Fold induction of CXCL13 in plasma	Fold induction of IL-6 In plasma
Age												
Sex (male)	-0.20 (0.21)											
Smoking	-0.13 (0.39)	**0.58** (<**0.01***)										
PS[a]	0.09 (0.56)	0.07 (0.66)	-0.08 (0.60)									
Chemotherapy	-0.18 (0.24)	-0.01 (0.97)	0.04 (0.82)	**-0.31** (**0.04***)								
Use of IS	0.10 (0.54)	0.05 (0.75)	**0.35** (**0.02***)	-0.01 (0.97)	-0.12 (0.43)							
irAE	-0.12 (0.44)	0.15 (0.32)	0.16 (0.30)	-0.07 (0.65)	0.20 (0.20)	0.06 (0.72)						
Pre % ICOS+ CD4+ T cells in PBMC	-0.20 (0.19)	-0.21 (0.16)	**-0.31** (**0.04***)	-0.13 (0.39)	0.13 (0.41)	-0.19 (0.22)	-0.08 (0.60)					
4wks % ICOS+ CD4+ T cells in PBMC	-0.17 (0.28)	-0.13 (0.41)	-0.10 (0.52)	-0.22 (0.15)	-0.03 (0.83)	**-0.20** (0.19)	0.23 (0.13)	**0.74** (<**0.01***)				
Δ % ICOS+ CD4+ T cells in PBMC	0.10 (0.50)	0.17 (0.27)	0.20 (0.20)	-0.08 (0.61)	-0.15 (0.32)	0.01 (0.93)	**0.37** (**0.01***)	-0.25 (0.10)	**0.38** (**0.01***)			
Fold induction of CXCL13 in plasma	-0.10 (0.52)	0.02 (0.91)	0.04 (0.81)	0.09 (0.55)	0.12 (0.43)	0.02 (0.91)	**0.34** (**0.03***)	-0.03 (0.84)	0.04 (0.82)	0.14 (0.36)		
Fold induction of IL-6 In plasma	-0.02 (0.88)	-0.15 (0.33)	0.03 (0.87)	**0.32** (**0.04***)	-0.13 (0.41)	0.03 (0.84)	-0.27 (0.08)	0.15 (0.33)	0.00 (0.98)	-0.23 (0.13)	0.25 (0.10)	

irAE, immune-related adverse event; PBMC, peripheral blood mononuclear cells; IS, immunosuppressants. The values given in parentheses indicate *p*-value.

[a] PS: performance status (0-3). * Statistically significant. TCS-IL6

Supplementary Table 3

Spearman's rank correlation matrix and the significance of relevant variables in the NSCLC patients with irAE pneumonitis and non-irAE

	Age	Sex (male)	Smoking	PS	Chemotherapy	Use of IS	irAE pneumonitis	Pre % ICOS+ CD4+ T cells in PBMC	4wks % ICOS+ CD4+ T cells in PBMC	Δ % ICOS+ CD4+ T cells in PBMC	Fold induction of CXCL13 in plasma	Fold induction of IL-6 In plasma
Age												
Sex (male)	0.06<br>(0.80)											
Smoking	0.18<br>(0.44)	**0.73**<br>(**<0.01***)										
PS[a]	0.09<br>(0.71)	0.12<br>(0.61)	-0.10<br>(0.69)									
Chemotherapy	-0.27<br>(0.26)	-0.13<br>(0.60)	0.03<br>(0.90)	**0.61**<br>(**<0.01***)								
Use of IS	0.13<br>(0.58)	0.35<br>(0.14)	0.42<br>(0.34)	-0.05<br>(0.84)	-0.22<br>(0.36)							
irAE pneumonitis	0.10<br>(0.68)	0.24<br>(0.32)	0.06<br>(0.81)	0.13<br>(0.59)	0.09<br>(0.71)	0.00<br>(1.00)						
Pre % ICOS+ CD4+ T cells in PBMC	-0.15<br>(0.52)	-0.45<br>(0.05)	**-0.47**<br>(**0.04***)	-0.20<br>(0.40)	-0.10<br>(0.69)	-0.40<br>(0.08)	-0.41<br>(0.08)					
4wks % ICOS+ CD4+ T cells in PBMC	0.08<br>(0.75)	-0.33<br>(0.16)	-0.38<br>(0.10)	-0.31<br>(0.18)	-0.02<br>(0.94)	-0.33<br>(0.16)	-0.02<br>(0.94)	**0.78**<br>(**<0.01***)				
Δ % ICOS+ CD4+ T cells in PBMC	0.41<br>(0.08)	0.27<br>(0.25)	0.11<br>(0.65)	-0.05<br>(0.83)	-0.27<br>(0.26)	0.35<br>(0.13)	**0.58**<br>(**<0.01***)	-0.32<br>(0.17)	0.15<br>(0.54)			
Fold induction of CXCL13 in plasma	-0.23<br>(0.34)	0.11<br>(0.64)	0.06<br>(0.80)	0.24<br>(0.30)	0.15<br>(0.52)	0.17<br>(0.47)	**0.46**<br>(**0.04***)	-0.34<br>(0.15)	-0.38<br>(0.10)	0.16<br>(0.49)		
Fold induction of IL-6 In plasma	0.05<br>(0.85)	0.01<br>(0.97)	0.26<br>(0.28)	0.40<br>(0.08)	0.00<br>(1.00)	-0.05<br>(0.83)	-0.20<br>(0.41)	-0.02<br>(0.94)	-0.26<br>(0.27)	-0.40<br>(0.08)	0.31<br>(0.18)	

irAE, immune-related adverse event; PBMC, peripheral blood mononuclear cells; IS, immunosuppressants. The values given in parentheses indicate *p*-value. [a] PS: performance status (0-3). * Statistically significant

Supplementary Table 4

Multivariate analysis association between irAE development and clinical variables in NSCLC patients

Variable	Coefficient	Odds ratio (95% CI)	*p* value
Δ % ICOS+CD4+ T cells in PBMC	0.61	1.83 (0.93 - 3.59)	0.08
Fold induction of CXCL13 in plasma	1.42	4.15 (1.08 - 15.95)	0.04
Constant	-0.69	0.50	0.04

CI, confidence interval.

Supplementary Table 5

Multivariate analysis association between development of irAE pneumonitis and clinical variables in NSCLC patients

Variable	Coefficient	Odds ratio (95% CI)	*p* value
Δ % ICOS+CD4+ T cells in PBMC	1.09	2.97 (0.89 - 9.91)	0.08
Fold induction of CXCL13 in plasma	2.29	9.88 (0.66 - 147.16)	0.10
Constant	-3.44	0.03	0.06

Propensity score = 1 / (1 + Exp (3.44 - 2.29×" Fold induction of CXCL13 in plasma" - 1.09×" Δ % ICOS+CD4+ T cells in PBMC")).

CI, confidence interval.

Supplementary Table 6

The sequence of primers for quantitative real-time PCR in this study		
Gene name	Primer sequence forward (5'-3')	reverse
Mouse *Il2*	GCGGCATGTTCTGGATTTGACTC	CCACCACAGTTGCTGACTCATC
Mouse *Il17a*	CTCAAAGCTCAGCGTGTCCAAACA	TATCAGGGTCTTCATTGCGGTGGA
Mouse *Il10*	GCTCTTACTGACTGGCATGAG	CGCAGCTCTAGGAGCATGTG
Mouse *Il22*	TGCTTCTCATTGCCCTGTG	TGGATGTTCTGGTCGTCACC
Mouse *Spp1*	AGCAAGAAACTCTTCCAAGCAA	GTGAGATTCGTCAGATTCATCCG
Mouse *Ccr5*	TGCACAAAGAGACTTGAGGCA	AGTGGTTCTTCCCTGTTGGCA
Mouse *Cx3cr1*	TGTCCACCTCCTTCCCTGAA	TCGCCCAAATAACAGGCC
